# A Systematic Review Comparing Four Transdiagnostic Programmes for School‐Age Children

**DOI:** 10.1002/cpp.70072

**Published:** 2025-05-06

**Authors:** Giorgio Ghizzoni, Maria Mirandi, Carlo Garofalo, Claudia Mazzeschi, Elisa Delvecchio

**Affiliations:** ^1^ Department of Philosophy, Social Sciences and Education University of Perugia Perugia Italy

**Keywords:** anxiety, children, depression, externalizing symptoms, internalizing symptoms, transdiagnostic programmes

## Abstract

Transdiagnostic programmes are useful for helping people with internalizing symptoms and comorbid psychopathology. These programmes have as their basic idea that disorders are not separate entities but share common aetiological and maintenance processes. Their use is important with children, who tend to present less differentiated psychopathological manifestations than adults. This systematic review aimed to examine the effectiveness of four evidence‐based transdiagnostic programmes: FRIENDS, Pyramid Club, Unified Protocol for Children (UP‐C) and Super Skills for Life (SSL). Studies from 2018 to 2023 including children between 4 and 14 years of age were examined. Following PRISMA guidelines, 48 studies that met the inclusion criteria were considered. Most of the studies referred to treatment, only a few used the selected transdiagnostic programmes for prevention, most notably FRIENDS and SSL. Very few of the studies reviewed have been randomized controlled trials, limiting the possibility to examine incremental benefits of these programmes over alternative approaches. The findings show that all programmes showed evidence of effectiveness, but UP‐C and SSL appear to be the most promising transdiagnostic programmes for the prevention and treatment of anxiety and depression in children as supported by more numerous studies in recent years. Moreover, UP‐C and SSL specifically appeared effective in reducing externalizing problems and improving social skills and prosocial behaviour. Although the results presented are promising, they should be regarded as provisional and in need of further support in randomized controlled trials to test the effectiveness of these evidence‐based programmes to ensure the psychological well‐being of children and thus reduce healthcare costs.

**Summary**

- Transdiagnostic programmes have shown clinical evidence in the treatment of internalizing and externalizing disorders.
- Transdiagnostic programmes are clinically relevant because they can be flexibly targeted at a range of emotional disorders and clinical comorbidities in children.
- We systematically reviewed the recent evidence of four transdiagnostic programmes for prevention and treatment in children: Unified Protocol for Children, Super Skills for Life, FRIENDS and Pyramid Club.
- Recent literature indicates that Unified Protocol for Children and Super Skills for Life programmes are the most effective in reducing symptoms transdiagnostically.
- These programmes are also important from a cost‐effectiveness perspective because they address fundamental processes common to several disorders within a single protocol.

## 1 Introduction

To date, scientific research has shown that symptoms occur along a continuum, highlighting the great likelihood of overlap between them, and that many disorders present comorbidity with each other, so it is limiting to categorize them (Fusar‐Poli et al. 2019; Roy‐Byrne 2017). Accordingly, transdiagnostic approaches to research and practice hold the basic idea that different disorders share common etiological and maintenance processes as well as cognitive‐affective, interpersonal and behavioural characteristics, best described by the latest studies on ‘factor p’, that is, a general factor underlying liability to psychopathology that cuts across diagnostic categories (Fusar‐Poli et al. 2019; Murray, Eisner, and Ribeaud 2016). The innovative aspect of transdiagnostic programmes is to work on common factors that underlie different manifestations of psychopathology, in a vision that goes beyond specific diagnoses. No particular problem is considered to fall into a specific diagnostic category, but specific techniques are used to address the difficulties that underlie internalizing and externalizing disorders and that impact personal development (Clark 2009). Transdiagnostic approaches are based on a dimensional view that makes no distinction between ‘normal’ and ‘pathological’ and, therefore, assumes that shared intervention goals can help prevent and treat the symptoms of psychopathology.

Levels of comorbidity are high in adult populations but are even higher in children and adolescents, where, for example, multiple diagnoses of anxiety make comorbidity the rule rather than the exception (Angold, Costello, and Erkanli 1999), and where youth with primary depressive disorders are at greater risk to show comorbid anxiety (Konac et al. 2021). Anxiety and depression are highly prevalent in childhood and adolescence and can generate many problems for the sufferer (Duduk 2022; World Health Organization 2023). A systematic review (Bor et al. 2014) reported that, in the 21st century, there have been clear increasing trends concerning anxiety and depressive disorders in children and adolescents. Because psychological problems, like anxiety and depression, tend to co‐occur in children and adolescents, interventions that target putatively transdiagnostic mechanisms hold promise to reduce the onset and recurrence of psychopathology (Fusar‐Poli et al. 2019). Accordingly, they intervene by improving the subject's emotional regulation capacity with strategies to be implemented to prevent or counteract the onset of symptoms (Essau et al. 2014).

The simultaneity aspect is one of the strengths of transdiagnostic treatments, as they allow for the promotion of multiple emotional domains, which is why it is crucial to ensure a cultural and economic investment in the growth of evidence‐based transdiagnostic programmes (Caiado et al. 2022). To date, a few evidence‐based transdiagnostic programmes for prevention and treatment in children have been developed, and their application to prevent or reduce psychopathology while improving children's social skills and well‐being represents an asset for mental health practice and public health more broadly. Because transdiagnostic approaches are based on different premises and use different techniques, an integrative overview of their characteristics and effectiveness evidence is crucial to aid policymakers in decision‐making (Craske 2012; Dalgleish et al. 2020).

To this end, the present study aimed to systematically review and compare some of the most popular transdiagnostic programmes for children between the ages of 4 and 14, to examine recent evidence in support of their effectiveness. Four transdiagnostic approaches were considered in the review as among the most used worldwide: FRIENDS programme, Pyramid Club, Unified Protocol for Children (UP‐C) and Super Skills for Life (SSL). The main features of each of these programmes are summarized in Table 1, and each of them is described below in specific subsections. These programmes originate with a common focus on anxiety/depression, but all have transdiagnostic aims to improve functioning more generally. The choice to include both clinical and nonclinical samples published in recent years (see Section 5) was motivated by the aim to include both prevention and treatment studies to provide the most recent evidence.

**TABLE 1:** Summary of the characteristics of the four transdiagnostic programmes.

Name of the programme	Number of sessions	Age of application	Application modality
FRIENDS programme	The programme consists of 10 to 12 sessions, each lasting 60 min.	The age of the participants in the intervention is between 4‐ and 15‐years old.	In‐person mode and in small groups
Pyramid Club	The intervention is delivered in 10 weeks with weekly 90‐min sessions.	The age of the participants in the intervention is between 7‐ and 14‐years old.	Intervention applied in groups of no more than 12 children
Unified Protocol	The programme is carried out with a qualified therapist for 15 weekly group sessions for 90′ at each meeting with the children.	The age of the participants in the intervention is between 7‐ and 14‐years old.	Intervention applied in groups or in an individual modality
Super Skills for Life	Applied in 8 sessions, delivered weekly, once or twice a week for 45 min per session.	The age of the participants in the intervention is between 6‐ and 11‐years old.	Intervention applied in groups with 6–8 children. Other applications are applied in an individual modality and in a computerized modality

### 1.1 FRIENDS Programmes

The FRIENDS programme is based on cognitive‐behavioural theories. It was created by Barrett (1998) and Barrett et al. (2015) in Australia, based on feedback from parents and children who initially participated in the first study of the group intervention programme. The FRIENDS programme implements various interventions to promote well‐being and prevent anxiety disorders and depression by working on protective factors such as resilience (Kozina 2021). The term FRIENDS constitutes an acronym, which is also useful for children to be able to remember the strategies they have been taught to be reused to handle difficult situations: F = Feelings (empathy training and self‐regulation); R = Remember to relax (relaxation and awareness strategies); I = I can do my best (changing useless thought to useful); E = Explore coping plans and strategies to find useful solutions (choosing thumbs‐up instead of thumbs‐down actions); N = Now reward yourself for doing your best (choose interpersonal rather than material rewards); D = Do not forget to practice (choose to use FRIENDS skills and give back to the community); S = Stay calm (value‐based role models and support networks). Thanks to these programmes, participants learn the skills to deal with their own emotions and those of others, problem‐solving, self‐confidence, resilience, self‐esteem and their quality of life.

There are four different types of FRIENDS programmes: Fun FRIENDS, FRIENDS for Life, My FRIENDS Youth and Adult Resilience. These programmes are specifically divided according to the age of the participants, thus Fun FRIENDS (4–7), FRIENDS for Life (8–9) and My FRIENDS Youth (10–15) (Pahl and Barrett 2007). Due to the scope of our review, we considered all three of these programmes but not the adult programme, and for convenience, we will always use the term ‘FRIENDS programme’ to refer to the three programmes. The FRIENDS programme lasts 10–12 sessions of 60 min each.

### 1.2 Pyramid Club

The Pyramid Club is a transdiagnostic intervention programme developed in the early 1980s (Syros, Karantzali, and Anastassiou‐Hadjicharalambous 2021). The programme to date is widely used in Anglo‐Saxon countries to prevent mental disorders in children aged from 7 to 14 years, improve peer relationships, emotional intelligence, pro‐social behaviour and reduce emotional problems (Cassidy 2014; McKenna, Cassidy, and Giles 2014). It is based on the theories of cognitive psychology and positive psychology and aims to help school children with internalizing disorders so that they can be guided towards healthy management of their difficulties and thus face the worries they experience with more self‐confidence. This type of intervention is delivered in 10 weeks with weekly 90‐min sessions in groups of no more than 12 children.

### 1.3 Unified Protocol

The Unified Protocol is a transdiagnostic intervention programme based on the theories of cognitive‐behavioural psychotherapy, developed by David Barlow and a group of researchers from the Center for Anxiety and Related Disorders (CARD) at Boston University (Barlow et al. 2010). It is a transdiagnostic approach targeting theory‐driven mechanisms shared by various psychological problems. Specifically, UP‐C aims to intervene on the functional processes responsible for emotion regulation, which are theorized in UP‐C as a fundamental aspect underlying psychopathology and influencing a wide range of outcomes, including positive and negative affect (Sakiris and Berle 2019). The children to whom UP‐C is addressed cover an age range of 7–13 years who present with emotional disorders of various kinds. This programme is very useful in preventing and counteracting the onset of anxiety and depression‐related disorders, increasing the ability of individuals who experience them to produce positive responses in dealing with internalizing problems (Barlow et al. 2017). The programme is carried out with a qualified therapist for 15 weekly group sessions of 90 min each.

### 1.4 SSL

SSL is a transdiagnostic intervention programme developed by Essau and Ollendick (2013) in England for children aged 6–11 years. Two further versions were developed during the years, one for adolescents aged 12–18 years (Essau and Ollendick 2019) and the other for young adults (Essau and Ollendick 2020). Like UP‐C, SSL is also grounded in a solid theoretical understanding of psychopathology. Specifically, SSL is based on the theories of cognitive‐behavioural psychotherapy and aims to increase self‐confidence, self‐esteem, the ability to respond positively to external difficulties, the regulation of emotional states and to be more aware of one's emotional states (both positive and negative) without judging them.

SSL aims to intervene in the mechanisms underlying emotion regulation that may be associated with internalizing and externalizing disorders (Diego, Morales, and Orgilés 2024b; Essau et al. 2014; Fernandes, Wright, and Essau 2023). There are eight sessions, delivered weekly, with groups of 6–8 children for 45 min per session. The sessions include role‐plays, homework, group and individual activities and video analysis. Each child receives a personal workbook, and each session ends with a specific homework assignment for the following week on a particular aspect that was addressed during the session.

## 2 Methods

**FIGURE 1:**
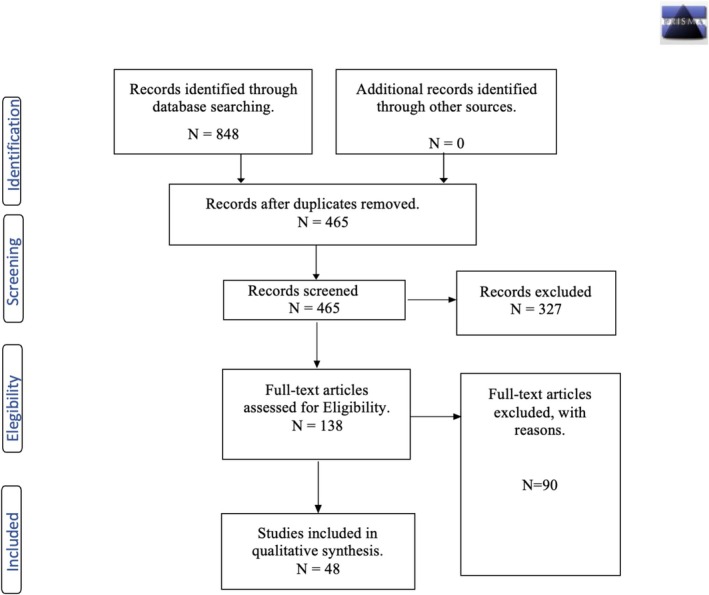
PRISMA flow diagram illustrating search strategy results and identifies of studies included in the review.

The present systematic review followed guidelines outlined in the Preferred Reporting Items for Systematic Reviews and Meta‐Analyses (Moher et al. 2009). The literature search was initially conducted in May 2023, with the aim to cover the past 5 years (i.e., filtering for studies published from 2018 onwards). The final search was conducted before the original submission on 13 December 2023, to include the most recent studies. Three electronic databases were employed: PubMed, Scopus and Web of Science. The search of articles was executed using specific keywords and Boolean logical operators to make the search as detailed as possible and to have an overview of the articles to be included and considered eligible. The present study focused on transdiagnostic interventions, which are FRIENDS programme, Pyramid Club, UP‐C and SSL, and so the selection of articles only considered these types of intervention. The following search terms were used: (‘FRIENDS PROGRAM*’ OR ‘PYRAMID CLUB’ OR ‘UNIFIED PROTOCOL’ OR ‘SUPER SKILLS FOR LIFE’) AND (‘TRANSDIAGNOSTIC’ OR ‘ANXIETY’ OR ‘DEPRESSION’ OR ‘PREVENTION’). Beyond transdiagnostic, we elected to specify ‘anxiety’ and ‘depression’ as search terms because all programmes reviewed in this study were originally developed with an emphasis on these highly prevalent conditions while preserving a transdiagnostic focus. In addition, we specified the term ‘prevention’ to cover a broader ground of studies not necessarily targeting clinical samples. Entering the search terms discussed above into the databases, several results were obtained, which were subjected to an initial screening phase based on the pre‐established inclusion and exclusion criteria. After the application of the search filters, the remaining articles were subjected to an additional screening phase by reading the abstracts, which made it possible to understand the eligibility of the articles (see Figure 1).

### 2.1 Inclusion and Exclusion Criteria

The inclusion criteria that were adopted are summarised below:

- Children aged between 4 and 14 years
- Articles in English language
- Publication of articles from the year 2018 to 2023
- Primary research studies (i.e., not reviews or meta‐analyses)
- Studies that included the four transdiagnostic programmes selected for research
- Studies with or without control condition.

All necessary information was extracted from each study deemed eligible, such as authors, year of publication, type of intervention, intervention results, sample size and age of participants.

## 3 Results

Applying the appropriate filters to the search, 848 articles were found in all three databases. The analysis phase of the studies was carried out via Rayyan, a web application for systematic reviews (Ouzzani et al. 2016).

After the removal of duplicates, 465 articles remained. After reading the abstracts and careful selection according to the inclusion/exclusion criteria, 327 articles were eliminated, and 138 full‐text eligible articles remained. By performing the reading of the full‐text during the screening phase, 90 articles were eliminated based on the inclusion/exclusion criteria. In total, 48 studies were included in the review.

Of the selected studies, 10 were randomized controlled trials (RCTs; Alaee et al. 2022; Diego, Morales, and Orgilés 2024a; Fernández‐Martínez et al. 2019; Fernández‐Martínez et al. 2021; Fjermestad et al. 2020; Fjermestad et al. 2022; Klein et al. 2021; Kozina 2021; Orgilés et al. 2023; Ramdhonee‐Dowlot, Balloo, and Essau 2021), while 16 were quasi‐experimental studies (Diego et al. 2024b; Fujisato et al. 2021; Gallegos‐Guajardo et al. 2020; Garcia et al. 2019; Jayman et al. 2019; Kozina 2018; Kozina 2020; Mehrdadfar et al. 2023; Melero et al. 2021; Melero et al. 2021; Melero et al. 2022; Nicolaidou et al. 2021; Orgilés et al. 2019; Orgilés, Espada, and Morales 2020; Orgilés et al. 2020; Orgilés et al. 2020). The rest employed a variety of designs (Table 2). Studies examined the effectiveness of the transdiagnostic programmes to varying degrees, highlighting the positive outcomes of each intervention on resilience, coping strategies, improved emotional regulation skills, emotional symptoms, empathic skills, improved prosocial behaviour and, in general, anxiety‐ and depression‐related symptoms. The main characteristics of each study are described below (Table 2). For ease of clarity, eligible articles are described here focusing on one programme at a time.

**TABLE 2:** Summary of the included studies.

Articles	Objective	Participants	Measures	1. Intervention 2. Design study	Results
**FRIENDS**					
Fisak et al. 2018	To evaluate the effectiveness of the FRIENDS programme as an early intervention for children with internalizing symptoms, combined with a resilience‐strengthening intervention focused on parents.	178 children (*M*_*age*_ = 5.27; *SD* = 0.93; 41.5% girls) participated in the study and completed the preintervention questionnaires. However, only 111 of these participants completed the postintervention questionnaires. Additionally, 100 mothers and 59 fathers provided complete data both before and after the intervention.	Measures assessing child functioning ‐ Preschool Anxiety Scale Parent report (Spence et al. 2001) to assess anxiety symptoms of the children 4–6 years old ‐ Spence Children's Anxiety Scale Parent report (Spence 1998) to assess anxiety symptoms of the children 6–18 years old ‐ Strengths and Difficulties Questionnaire—Parent report (Goodman 1997) to assess behavioural difficulties and competencies ‐ Children's Depression Inventory (Kovacs 1992) to assess depressive symptomology Measures of parent functioning ‐ Depression Anxiety and Stress Scales—Short form (Lovibond and Lovibond 1995) to discriminate the main symptoms of depression, anxiety and stress and identify the locus of emotional disturbance ‐ Parenting Stress Index Short form (Abidin 1995) to assess levels of stress within the parent child relationship ‐ Devereux Adult Resilience Survey (Ball and Mackrain 2009) to assess the perceptions of one's own personal strengths	1.Treatment 2.Open trial	From preintervention to postintervention comparison, significant decreases were found in anxiety for both younger (*t* = 4.52, *p* < 0.001) and older children (*t* = 7.85, *p* < 0.001), as well as in depression in the total sample (*t* = 4.12, *p* < 0.001). Significant improvement was also observed in the total difficulties (*t* = 4.99, *p* < 0.001). In addition, after the intervention also both mothers and fathers showed significant decreases in their own levels of anxiety and depression (*t* = 3.91, *p* < 0.001; *t* = 3.42, *p* < 0.01), parenting stress (*t* = 8.07, *p* < 0.001; *t* = 2.38, *p* < 0.05) and significant increases in the level of resilience (*t* = 4.51, *p* < 0.001; *t* = 3.26, *p* < 0.01).
Fjermestad et al. 2020	1. Compare levels of symptoms at baseline (i.e., anxiety, depression and conduct problems) between the targeted prevention school sample and the clinical sample. 2. Examine changes in symptoms from baseline to posttreatment and 3‐ and 12‐month follow‐up within the targeted prevention school sample to provide initial evidence for FRIENDS as targeted prevention in Norway. 3. Compare the results of the targeted prevention school sample to the results of the clinical sample.	The targeted prevention sample consisted of 82 participants (*M_age_* = 11.6; *SD* = 2.1; 75.0% girls), while the clinical sample with anxiety disorders included 88 participants (*M_age_* = 11.7, *SD* = 2.1; 54.5% girls).	‐ Spence Children's Anxiety Scale (Spence 1998) child and parent versions were used to assess anxiety symptoms ‐ Short Moods and Feelings Questionnaire (Angold et al. 1995) child and parent versions were used to assess the depressive symptoms ‐ Strengths and Difficulties Questionnaire (Goodman 1997) child and parent versions were used to assess behavioural difficulties and competencies	1. Prevention and treatment 2.Randomized controlled trial	Both children and their parents found no significant differences between the prevention sample and clinical samples on any scale, except for anxiety symptoms, where parents reported a higher level in the clinical sample (*p* < 0.001). Regarding anxiety symptoms, there was a significant reduction from pre to posttreatment for both in reports from the youth themselves (*p* < 0.001) and from their parents (*p* < 0.01). There was no significant reduction from posttreatment to follow‐up at 3 months. However, there was a significant reduction of anxiety and conduct problems in parent‐reported symptoms (*p* < 0.001), but not youth, from 3‐ to 12‐months follow‐up.
Fjermestad et al. 2022	Examine whether exposure to feared situations is associated with outcomes measured at posttreatment and 1‐year follow‐up.	68 participants *(M*_*age*_ = 11.1; *SD* = 2.1, 45.5% girls) and 17 therapist (*M* = 49.8; *SD* = 9.4; 93.3% girls).	‐ Anxiety Disorders Interview Schedule/Clinical Severity Ratings (Silverman and Albano 1996) child and parent versions were used to determine diagnostic status ‐ Spence Children's Anxiety Scale (Spence et al. 2001) child and parent versions were used to assess anxiety symptoms ‐ Therapeutic Alliance Scale for Children (Shirk and Saiz 1992) was used to assess alliance ‐ Quality of the Exposure Component Form (Fjermestad et al. 2022) it is rated by independent observers	1. Treatment 2. Randomized controlled trial	Parental contribution at the end of each session was significantly associated with the decrease in all anxiety diagnoses at 1‐year follow‐up (*β* = 3.24; *p* < 0.05). In contrast, none of the other variables were associated with outcomes at 1‐year follow‐up (*β* = 1.63; *p* < 0.001). Finally, in terms of dropout, tests showed that the preparedness and total quality of exposure to feared situations were significantly lower for the two dropout cases (*p* < 0.05).
Gallegos‐Guajardo et al. 2020	1. To evaluate the effectiveness of the FRIENDS programme in reducing anxiety symptoms in children and improving social and emotional skills, as assessed by parents. 2. To evaluate the effectiveness of the programme on difficulties and prosocial behaviour, as evaluated by parents and teachers.	49 children (*M*_*age*_ = 6.55; *SD* = 0.50; 51% boys) participated, with their parents and two teachers.	‐ Preschool Anxiety Scale Parent report (Spence et al. 2001) was used to assess anxiety symptoms of their children ‐ Behavioural and Emotional Rating Scale Parent report (Epstein 2010) was used to assess the emotional and behavioural strengths of their children ‐ Strengths and Difficulties Questionnaire (Goodman 1997) was completed by both parents and teachers to assess behavioural difficulties and competencies of the children	1. Treatment 2. Quasi‐experimental study	The results showed a statistically significant decrease in separation anxiety symptoms (*t* = 3.25; *p* < 0.01), and an increase in affective (*t* = 7.78; *p* < 0.001), intrapersonal and interpersonal strengths (*t* = 3.54; *p* < 0.001; *t* = 2.38; *p* < 0.01) and prosocial behaviour (*t* = 2.63; *p* < 0.01).
Garcia et al. 2019	To evaluate the effectiveness of FRIENDS as an intervention for children with internalizing symptoms by promoting the development of social emotional skills.	25 children (*M*_*age*_ = 6.55; 72% boys) participated, with their parents.	‐ Child Behaviour Checklist Parent report (Achenbach and Rescorla 2001) was used to assess social skills and behavioural problems of their children ‐ Strengths and Difficulties Questionnaire Parent report (Goodman 1997) was used to assess behavioural difficulties and competencies of their children ‐ Spence Children's Anxiety Scale Parent report (Spence 1998) was used to assess anxiety symptoms of their children	1. Treatment 2. Quasi‐experimental study	Statistically significant reduction in posttreatment was found in the anxiety subscales (*Z* = 3.13; *p* < 0.01), depression (*Z* = 1.99; *p* < 0.05), somatic complaints (*Z* = 2.05; *p* < 0.05), social problems (*Z* = 3.56; *p* < 0.001) and emotional symptoms (*Z* = 2.93; *p* < 0.01). In addition, anxiety and emotional symptoms were also significantly reduced from posttreatment to follow‐up (*Z* = 2.44; *p* < 0.01; *Z* = 3.04; *p* < 0.01). Focusing on the externalizing difficulties, statistically significant reductions of symptoms were observed for aggressive behaviour (*Z* = 3.93; *p* < 0.001 from pretreatment to follow‐up), violation of rules (*Z* = 3.47; *p* < 0.001 from pretreatment to follow‐up), hyperactivity (pre/post, *z* = 2.96; *p* < 0.05 and pre/follow, *z* = 1.93; *p* < 0.05), conduct problems (pre/post, *Z* = 1.95, posttest; *p* < 0.05 and pre/follow, *Z* = 2.07; *p* < 0.05) and in the total scale of externalizing problems (*Z* = 3.99; *p* < 0.001). In general, there was a reduction of the total difficulties in comparing pre and posttreatment moments (*Z* = 2.36; *p* < 0.01) and pre‐treatment and follow‐up moments (*Z* = 3.10; *p* < 0.01).
Hosokawa et al. 2023	To evaluate the effectiveness of the FRIENDS programme for improving social skills in children.	94 participants (*M*_*age*_ = 4.72; *SD* = 0.33; 45.45% girls) included in the intervention group and 66 participants in the control group (*M*_*age*_ = 4.82, *SD* = 0.33; 45.45% girls).	‐ Social Skills Questionnaire for Preschoolers Teacher report (Takahashi et al. 2008) was used to assess the social skills of the children	1. Prevention 2. Cohort study	The results showed significantly higher scores of self‐control (*p* < 0.05) and cooperation (*p* < 0.001) in the intervention group compared to the control group.
Klein et al. 2021	1. To assess whether parents of children with social anxiety reported different levels of social skills between pretreatment with the FRIENDS programme and posttreatment than parents of children without anxiety disorders. 2. To assess whether levels of social skills predicted treatment outcome. 3. To assess whether there was an interaction between parent‐reported social skills and social anxiety.	124 participants (*M*_*age*_ = 10.10; *SD* = 1.27; 59 girls), including 39 with Social Anxiety Disorder and 85 without. Of which 65 were treated individually and 59 were treated in groups. In addition, the following were involved 123 mothers and 108 fathers.	‐ Multidimensional Anxiety Scale for Children (March et al. 1997) a measure of paediatric anxiety ‐ Child Behaviour Checklist Parent report (Achenbach 1991) was used to assess emotional and behavioural problems of their children ‐ Social Skills Rating System Parent report (Gresham and Elliott 1990) was used to assess the social skills of their children ‐ Anxiety Disorders Interview Schedule (Silverman and Albano 1996) child and parent versions were used to determine diagnostic status	1.Treatment 2. Randomized controlled trial	Comparison of pretreatment parent‐reported social skills in children with and without Social Anxiety Disorder showed lower levels of assertiveness *t*(120) = 5.06, *p* < 0.001 and responsibility *t*(120) = 5.08, *p* < 0.001 in those with Social Anxiety Disorder. The results showed that higher levels of pretreatment assertiveness and self‐control reported by parents were significantly linked to a more favourable outcome in both parents' perceptions of their children's internalizing problems (mother: *β* = 0.18, *p* < 0.05; *β* = 0.29, *p* < 0.01; father: *β* = 0.30, *p* < 0.05; *β* = 0.42, *p* < 0.05).
Kozina 2018	To evaluate, through a quasi‐experimental study, the effectiveness of the FRIENDS programme as a treatment for children with externalizing difficulties.	73 children participated (grade 8 aged 13–14 years) in the study, including 38 in the intervention group (52.63% girls) and 35 participants in the control group (54.29%).	‐ AGUD Aggression scale (Kozina 2013) to assess the aggression ‐ Strengths and Difficulties Questionnaire (Goodman 1997) to assess behavioural difficulties and competencies	1.Prevention 2. Quasi‐experimental study	Results showed that in the intervention group, levels of general aggression (and specific forms of aggression) were similar before and after the programme, while in the control group, there was an increase in general aggressiveness (*F* = 3.79, *p* < 0.05) and aggression against authority (*F* = 8.08, *p* < 0.01) from premeasurement to postmeasurement. In addition, time effects on the difficulties of externalization (*F* = 8,22, *p* < 0.001), on the problems of conduct (*F* = 2,92, *p* < 0.05) and on hyperactivity (*F* = 7.75, *p* < 0.001) were observed in the intervention group whereas in the control group, there was no timeout effect on outsourcing difficulties.
Kozina 2020	To evaluate the effectiveness of the FRIENDS programme as an intervention in adolescents in reducing internalizing difficulties.	The experimental group included 44 participants (52.27% girls), while the control group consisted of 36 adolescents (55.56% girls) aged between 13 and 14 years (grade 8)	‐ ANUD Lestvica agresivnosti za učence in dijake (Kozina 2012) to assess the general anxiety ‐ Strengths and Difficulties Questionnaire (Goodman 1997) to assess behavioural difficulties and competencies	1.Prevention 2. Quasi‐experimental study	At the postintervention assessment, the intervention group reported lower overall anxiety (*F* = 7.44, *p* < 0.01) and worries (*F* = 9.55 *p* < 0.05), persisted at the 6‐month follow‐up, although the effects were smaller than at the postintervention assessment. In addition, a main effect of time was found on internalizing difficulties (*F* = 7.27, *p* < 0.001) and with respect to relationship with peers (*F* = 15.18, *p* < 0.001).
Kozina 2021	To evaluate the effectiveness of the FRIENDS programme as an intervention in reducing levels of anxiety and aggression in schoolchildren.	The experimental group included 44 participants (54.55% girls), while the control group included 41 participants (41.46% girls) aged 9–10 years old (4th grade).	‐ ANUD Lestvica agresivnosti za učence in dijake (Kozina 2012) to assess the general anxiety and the three anxiety components: emotions, worries and decisions ‐ AGUD Aggression scale for pupils and students (Kozina 2013) to assess the aggression	1.Prevention 2. Randomized control study	Results showed decreased levels of anxiety (*F* = 3.97, *p* < 0.01) and its components: emotions (*F* = 5.95, *p* < 0.001) and decisions (*F* = 3.09, *p* < 0.05) from before measurement to follow‐up. Additionally, the repeated measures ANOVA showed that aggression toward authority changed significantly over time (*F* = 6.56, *p* < 0.001).
Nicolaidou et al. 2021	1. Evaluate the extent to which interactive resilience learning environment, based on the FRIENDS programme, is effective in reducing anxiety in children. 2. Investigate to what extent is the interactive learning environment, based on the ‘Friends’ prevention programme, for resilience effective in increasing children's ability to identify emotions and symptoms of stress and to remember stress management techniques. 3. To investigate how children evaluate the usability of the interactive learning environment for resilience, based on the FRIENDS prevention programme.	20 participants included in the intervention group (55% boys) and 21 in the control group (47.62%) aged 9–10 years old (4th grade)	‐ Spence Children's Anxiety Scale ( Spence 1998 ) to assess anxiety symptoms ‐ Open‐ended questions to assess the ability to recognize emotions and symptoms of anxiety and to remember ways to manage stress System Usability Scale to measure the usability of the learning environment	1.Prevention 2. Quasi‐experimental study	After the intervention, there was a small but nonsignificant reduction in reported anxiety symptoms, with the exception of obsessive‐compulsive symptoms in the intervention group (*t* = 5.16; *p* < 0.01). In addition, the intervention group showed a significant increase in the ability to identify basic emotions (*t* = 6.99; *p* < 0.001), a reduction in somatic symptoms (*t* = 7.31; *p* < 0.001) and an improvement in stress management (*t* = 6.85; *p* < 0.001).
Van Der Mheen et al. 2020	To evaluate the effectiveness of the FRIENDS programme as an intervention for children with anxiety disorders by identifying predictors of treatment progress.	28 participants (*M*_*age*_ = 6.6; *SD* = 1.1; 57.1% boys) with anxiety disorders.	‐ Anxiety Disorders Interview Schedule for Children (Siebelink and Treffers 2001) was used to determine diagnostic status ‐ Child Behaviour Checklist Parent report (Achenbach and Rescorla 2001) to assess emotional and behavioural problems in their children	1.Treatment 2. Open trial study	A significant decrease was found for emotional and behavioural problems (*t* = 2.38, *p* < 0.05), internalizing problems (*t* = 2.32, *p* < 0.05) and for anxiety problems (*t* = 2.17, *p* < 0.05). In addition, the level of preintervention anxiety problems significantly predicted treatment progress (*β* = 0.537, *F* = 5.27, *p* < 0.05, *R* ^2^ = 0.29).
**Pyramid Club**					
Jayman et al. 2019	1. To evaluate the effectiveness of the Pyramid programme in children in levels of social emotional wellbeing. 2. To assess, through investigation of the perceptions and experiences of service users and programme leaders, an in‐depth understanding of the effectiveness of the programme.	66 participants included in the intervention group and 60 in the control group (*M*_*age*_ = 12.53; *SD* = 0.79; 58.73% girls).	‐ Strengths and Difficulties Questionnaire (Goodman 1997) was completed by children and class teachers to assess behavioural difficulties and competencies	1.Treatment 2. Quasi‐experimental study	A significant decrease in total difficulties for the Pyramid group (*t* = 7.62, *p < 0*.001). Specifically, subscale analysis demonstrated significant changes over time in three domains: emotional symptoms (*F* = 22.73, *p < 0*.001), relationship problems with peers (*F* = 28.37, *p < 0*.001) and in prosocial behaviour (*F* = 5.46, *p* < 0.01). At 12‐months follow‐up, children showed a significant difference between T1 and T3 scores for the Pyramid group (*t* = 7.47, *p < 0*.001), demonstrating consistent results on two subscales: emotional symptoms (*t* = 6.04, *p < 0*.001) and relationship problems with peers (*t* = 7.47, *p < 0*.001).

Alaee et al. 2022	To evaluate the effectiveness of the UP‐C as a transdiagnostic treatment in children with emotional disturbance by reducing levels of anxiety sensitivity, negative affect and perceived control.	The 34 participants within the study (aged 7 to 13) with emotional disorders were randomly assigned within the treatment group, consisting of 18 participants (*M_age_* = 10.22, *SD* = 1.96; 77.8% girls), and the control group, consisting of 16 participants (*M_age_* = 10.13, *SD* = 2.09; 56.3% girls).	‐ Anxiety Disorders Interview Schedule for DSM‐IV Child and Parent Version (Storch et al. 2012) is a semistructured diagnostic interview to assess the presence, nature and severity of anxiety, depression and externalizing disorders ‐ Revised Children's Anxiety and Depression Scale Parent Report (Chorpita, Moffitt, and Gray 2005) to assess the severity of emotional disorders' symptoms ‐ Child Symptom Inventory Parent Report (Verhulst and van der Ende 2006) to assess emotional disorders' symptoms ‐ Positive/Negative Affect Schedule for Children (Ebesutani, Regan, et al. 2012) to assess the positive and negative affect of children ‐ Children's Anxiety Sensitivity Index (Silverman et al. 1991) to assess fear of somatic sensations, fear of cognitive dyscontrol and fear of socially observable anxiety symptoms ‐ Anxiety Control Questionnaire Children (Weems 2005) to assess children's perceptions of how much control they have over both internal and external stimuli	1.Treatment 2..Randomized controlled trial	The results indicated that, in the treatment group (UP‐C), levels of negative affect significantly decreased compared to the control group, both in the posttreatment period (*F* = 21.83, *p* < 0.001) and at the 3‐month (*F* = 28.44, *p* < 0.001) and 8‐month follow‐up (*F* = 48.99, *p* < 0.001). Additionally, in the treatment group, there was a significant reduction in the physical (*F* = 15.71, *p* < 0.001) and mental (*F* = 12.12, *p* < 0.001) components of Anxiety Sensitivity from pretreatment to the follow‐up. At the 8‐month follow‐up, a significant difference also emerged in perceived control scores, for both internal (*F* = 29.51, *p* < 0.001) and external dimensions (*F* = 32.17, *p* < 0.001).
Burton et al. 2021	To evaluate the effectiveness of the family‐based treatment (FBT) approach incorporated to UP‐C for avoidant/restrictive food intake disorder (ARFID) as a treatment in children with ARFID and autism spectrum disorder (ASD), in comorbidity.	Two girls: Lauren 6‐years old and Rachel 11‐years old.	No measures have been proposed.	1.Treatment 2. Case study	The application of the FBT + UP‐C protocol appears to improve food intake, increase food variety and reduce reliance on nasogastric feeding, while also reducing anxiety symptoms related to oral intake in the two cases presented. For Rachel, the UP‐C protocol enhanced emotional awareness, strong emotion management and distress tolerance while reducing anxiety sensitivity through interoceptive exposure and cognitive reappraisal. Her mother noted improved self‐regulation in anxiety‐inducing situations. For Lauren, learning about emotional response components, the avoidance cycle and present moment awareness helped her reach treatment goals. The progress of both Rachel and Lauren supports UP‐C as an effective transdiagnostic model adaptable to diverse treatment needs.
Caiado et al. 2022	To assess the acceptability and feasibility of UP‐C and their parents and preliminary effects on treatment outcomes by analysing levels of internalizing symptoms, severity of alimentary disorders and interference in the lives of children and parents.	32 children (*M*_*age*_ = 8.25; *SD* = 1.83; 48% girls) with emotional disorders and their parents (*M* = 40.78; *SD* = 3.62).	‐ Mini International Neuropsychiatric Interview for Children and Adolescents (Sheehan et al. 2010) child and parent report were used to assess the presence of mental illness ‐ Revised Children's Anxiety and Depression Scale (Ebesutani, Reise, et al. 2012) child's report to assess children's anxiety and depression symptoms ‐ Children's Anxiety and Life Interference Scale (Lyneham et al. 2013) child and parent report were used to assess the life interference and life impairment of the main symptomatology experienced by the child	1.Treatment 2. Single‐armed design	The results showed significant differences in children's global functioning at pretreatment and posttreatment (*Z* = 4.85; *p* < 001). A significant effect over time was found in the overall infant anxiety and depression scales (*F* = 29.50, *p* < 0.001) and for anxiety scores (*F* = 35.45, *p* < 0.001), as well as for the life interference scales for children (*F* = 52.95, *p* < 0.001) and for parents (*F* = 14.03, *p* < 0.001). Finally, for effects at 3‐month follow‐up, statistically significant differences were found between pretreatment and follow‐up and between midtreatment and follow‐up for all variables.
Ebrahimi et al. 2019	1. To evaluate the effectiveness and applicability of the UP‐C for children with emotional disorders. 2. To examine the effectiveness of the UP‐C compared with cognitive behavioural therapy (CBT).	45 girls (aged 8 to 11 years) with an internalizing disorder were randomly divided into three groups. The UP‐C group (*M*_*age*_ = 9.13; *SD* = 1.02), the CBT group (*M*_*age*_ = 9.27, *SD* = 1.02) and the control group (*M*_*age*_ = 9.20, *SD* = 0.98). Each group consisted of 15 participants.	‐ Kiddie Schedule for Affective Disorders and Schizophrenia and lifetime version (Birmaher et al. 2009) to assess the psychopathological disorders ‐ Revised Children's Anxiety and Depression Scale (Ebesutani, Reise, et al. 2012) to assess the psychological disorders	1.Treatment 2. Semi‐experimental research	The results showed a significant difference between pretreatment and posttreatment in the UP‐C group and CBT group (*F* = 6.14, *p* < 0.01). In addition, in both treatment groups, there was a significant difference between pretreatment and follow‐up (*F* = 5.22, *p* < 0.01). Specifically, there was a decrease in posttreatment depressive anxiety (*F* = 5.69, *p* < 0.05).
Eckhardt et al. 2019	To examine, through a case study, the effectiveness of a combined approach of Family Based Treatment (FBT) and UP‐C in a child with avoidant/restrictive food intake disorder (ARFID).	One participant, a 9‐year‐old girl, diagnosed ARFID, anxiety disorder and specific choking phobia.	No measures have been proposed.	1.Treatment 2.Case study	This case study demonstrates that the FBT + UP‐C therapy model for ARFID proved feasible and effective in helping the patient gain weight, return to eating a variety of foods in different settings and reduce anxiety related to eating and separation from her mother. At follow‐up, 5 months after completing treatment, the patient's weight continued to increase and was able to separate and eat away from her mother without significant difficulty.
Dimitropoulos et al. 2023	To evaluate the adaptation of UP‐C within a residential treatment facility for children with experiences of child maltreatment.	19 participants (aged 9–13 years) and their parents, and 20 staff members, who participated in the focus groups.	Qualitative measurements Two focus groups and three individual interviews were conducted with 20 staff members. Quantitative measurements ‐ Screen for Child Anxiety‐related Disorders (Birmaher et al. 1997) was administered to the child, the caregiver and a staff member to assess the anxiety ‐ Child Depression Inventory 2 Short Form (Kovacs 2011) was administered to the child, the caregiver and a staff member to assess the depression	1.Treatment 2. Qualitative study	Qualitative analysis showed that staff perceived UP‐C as compatible with the needs of the children in their care. Many children showed a reduction in anxiety symptoms over the course of treatment (42% according to child self‐report; 21% from caregiver report; 70% from staff report). Similarly, children often showed a reliable reduction in depression symptoms (37% from child self‐report; 21% from caregiver report; 60% from staff report). In terms of caregiver self‐reported mental health, 70% showed reduction in emotion dysregulation.
Fujisato et al. 2021	1. To evaluate the preliminary effectiveness of UP‐C as a treatment in children with emotional disorders. 2. To develop the Japanese version of UP‐C, evaluating acceptability and feasibility.	17 participants (aged 8 to 12; *M*_*age*_ = 10.06; *SD* = 0.97; 64.71% girls) with emotional disorders.	‐ Clinical Global Impression Improvement Scale (Guy 1976) to assess the emotional disorders ‐ Spence Children's Anxiety Scale (Spence 1998) to assess the anxiety symptoms ‐ Depression Self‐Rating Scale for Children (Birleson 1981) child and parent report to assess the depression ‐ Child Outcome Rating Scale (Duncan et al. 2006) child and parent report to assess the functional status ‐ Client Satisfaction Questionnaire (Larsen et al. 1979) child and parent report to assess the treatment satisfaction	1. Treatment 2. Quasi‐experimental study	Anxiety improved significantly at posttreatment (child report: *MD* = 15.63, 95% CI = 25.07 to 6.20, *p* < 0.001; parent report: *MD* = 13.35, 95% CI = 23.05 to 3.66, *p* < 0.01) and at 3‐month follow‐up (child report: *MD* = 23.82, 95% CI = 33.26 to 14.38, *p* < 0.001; parent report: *MD* = 25.35, 95% CI = 35.05 to 15.66, *p* < 0.001) compared with pretreatment in both child and parent reports. In addition, the children's multifaceted functional status improved gradually during the study period and were significantly higher at the 3‐month follow up than pretreatment (*MD* = 5.95, 95% CI = 0.3711.53, *p* < 0.05). Parents reported similar scores at midtreatment compared with pretreatment (*MD* = 7.30, 95% CI = 2.0012.61, *p* < 0.01), and this treatment effect was maintained during posttreatment (*MD* = 6.86, 95% CI = 1.5612.17, *p* < 0.01) and the 3‐month follow‐up period (*MD* = 7.09, 95% CI = 1.7912.39, *p* < 0.01).
Grossman and Ehrenreich‐May 2020	1. To present the application of UP‐C in children with frequent and severe outbursts of anger and/or irritability. 2. Demonstrate the adaptation of the treatment to deal with anger in children with emotional disorders.	One participant, Zara, 8‐years old with emotional problems.	‐ Children's Emotion Management Scale–Anger (Zeman, Shipman, and Penza‐Clyve 2001) to assess the strategies used to regulate emotions	1.Treatment 2. Case study	UP‐C has been effectively applied to address anger and the emotional behaviours associated with it. Zara has learned effective strategies for managing emotions and has found ways to modify her automatic and maladaptive reactions. During treatment, both Zara and her mother reported a significant reduction in symptoms. The evaluations of the main problems, provided by Zara and her mother, showed remarkable improvement, and the treatment gains were reflected in Zara's scores on emotion management.
Góis et al. 2022	To evaluate the application of the UP‐C in a child with comorbid anxiety symptoms.	Only one participant, B., an 8‐year‐old girl, with comorbid anxiety disorders (e.g., separation anxiety, specific phobia [dogs], agoraphobia and social anxiety).	‐ Revised Child Anxiety and Depression Scale (Chorpita, Moffitt, and Gray 2000) to assess children's anxiety and depression symptoms ‐ Child Anxiety Life Interference Scale for children and parents (Lyneham et al. 2013) to assess the life interference and impairment of the core symptomatology	1. Treatment 2. Case study	This case study highlights the clinical utility of UP‐C in reducing symptoms of anxiety and depression, as well as improving overall functioning and emotional symptoms. After the implementation of the programme, B. was able to be in the car and at home without her mother, interact with dogs, try new activities and present at school with greater confidence and assurance. These improvements were sustained even after 3 months.
Hawks et al. 2020	1. To examine the effectiveness, flexibility and acceptability of an adapted version of the UP‐C as a treatment in children with persistent irritability and/or disruptive behaviour. 2. To examine the effectiveness of an adapted version of UP‐C as a treatment in children in the mechanisms deputed to symptoms of irritability and/or disruptive behaviours, such as emotion regulation, information processing bias; problems with peers. 3. To examine the effectiveness of treatment in changing emotional symptoms.	19 participants (*M*_*age*_ = 10.01; 78.90% boys) with primary presenting concerns of irritability and/or disruptive behaviours. Children participated in the programme with at least one parent.	‐ Affective Reactivity Index (Stringaris et al. 2012) to assess the irritable mood ‐ Disruptive Behaviour Rating Scale Parent report (Barkley 1997) to assess the oppositional defiant disorder, and conduct disorder ‐ Emotion Regulation Checklist Parent report (Shields and Cicchetti 1997) to assess the emotion regulation ‐ Strengths and Difficulties Questionnaire Parent report (Goodman 1997) to assess behavioural difficulties and competencies ‐ Children's Automatic Thoughts Scale (Schniering and Rapee 2002) to assess negative self‐statements	1. Treatment 2. Interventional pilot study	Parents' ratings of their children's irritability (*t* = 4.84, *p* < 0.001), oppositional behaviours (*t* = 3.14, *p* < 0.01), emotional problems (*t* = 2.38, *p* < 0.05), behavioural problems (*t* = 3.19, *p* < 0.01) and conduct problems (*t* = 3.21, *p* < 0.01) were significantly lower at posttreatment than at baseline.
Kennedy, Bilek, and Ehrenreich‐May 2019	1. To evaluate the efficacy of UP‐C compared to a specific anxiety‐focused treatment protocol as a control condition (Cool Kids; CK). 2. Examine condition‐related differences in remission and diagnostic response as assessed by the clinician, and anxiety and depression symptoms assessed by the parents and child. 3. Examine condition‐related differences in emotional regulation variables as assessed by parents and children, as maintenance factors for emotional disorders.	47 participants (*M*_*age*_ = 9.3*;* 55.31% girls), randomly divided into 24 participants included in the UP‐C and 23 participants assigned to the active comparison, with at least one primary anxiety disorder.	‐ Anxiety Disorders Interview Schedule for DSMIV (Silverman and Albano 1996) child and parent versions were used to determine diagnostic status ‐ Clinician Global Impression–Improvement Scale (Guy 1976) to assess the severity of all emotional disorder diagnoses ‐ Screen for Child Anxiety Related Emotional Disorders (Birmaher et al. 1997) child and parent report to assess the anxiety ‐ CDI—Child and Parent Reports (Kovacs 1992) child and parent report to assess the depressive symptoms ‐ Children's Emotion Management Scales parent version (Zeman et al. 2010) to assess the regulating emotions ‐ Emotion Regulation Questionnaire (Gullone and Taffe 2012) to assess the cognitive and expressive suppression	1.Treatment 2. Randomized controlled pilot trial	The results did not show any differences in the remission of the primary diagnosis between children enrolled in the UP‐C programme and those in the CK programme after treatment (61.90% vs. 57.90%, χ^2^ = 0.067, *p* = 0.80, OR = 1.18). There was also no significant difference in the likelihood of achieving remission of their primary diagnosis between the UP‐C and CK groups (70.60% vs. 63.60%, χ^2^ = 0.15, *p* = 0.70). Furthermore, participants in the UP‐C and CK groups did not significantly differ in their likelihood of achieving remission from all emotional disorder diagnoses at follow‐up (64.70% vs. 54.50%, χ^2^ = 0.29, *p* = 0.59).
Kennedy et al. 2018	To examine whether depressive symptoms, social anxiety, anxiety severity and parental psychopathology predict the treatment outcome of the UP‐C in paediatric populations.	79 participants (*M*_*age*_ = 9.47; *SD* = 1.68; 53,30% girls) with a primary anxiety diagnosis (with or without comorbid depression). Of these, 60 participants were considered treatment completers, having data available for analysis at posttreatment.	‐ Anxiety Disorders Interview Schedule for the DSMIV Child and parent reports (Silverman and Albano 1996) was used to determine diagnostic status ‐ Clinician Global Impression Severity Scale (Guy 1976) to assess the severity of all emotional disorder diagnoses ‐ Screen for Child Anxiety Related Emotional Disorders (Birmaher et al. 1997) child and parent report to assess the anxiety ‐ Depression, Anxiety and Stress Scales Parent report (Lovibond and Lovibond 1995) to assess symptoms of depression, anxiety and physiological arousal/stress ‐ Penn State Worry Questionnaire—Parent report (Meyer et al. 1990) to assess pathological worry	1. Treatment 2. Observational study with a cohort approach	The results showed that 51.67% of patients achieved remission of all emotional disorder diagnoses during posttreatment. Specifically, there was a statistically significant difference in posttreatment in general anxiety (*β* = 0.06, *p* < 0.05), social anxiety (*β* = 0.17, *p* < 0.05) and externalizing symptoms (*β* = 9.11, *p* < 0.001). In addition, general anxiety (*β* = 0.089, *p* < 0.001) and social anxiety (*β* = 0.178, *p* < 0.001) assessed by the child at pretreatment were predictive of a higher likelihood of achieving a reliable change in anxiety assessed by the child over the course of treatment.
Kennedy, Halliday, and Ehrenreich‐May 2021	1. To investigate the presence of distinct classes of response to the UP‐C using self‐reported and parent‐reported anxiety and depression scores. 2. To examine whether demographic variables (age and gender) and variables associated with poor/differential response in previous studies (overall severity, presence of depression diagnosis, social anxiety or obsessive‐compulsive disorder) predicted the likelihood of belonging to a particular response class. 3. Identify the percentage of decrease in anxiety and depression scores at midtreatment that best discriminated between responders and nonresponders.	33 children (*M*_*age*_ = 13.36; SD = 3.36; 56.40 girls) of whom 20 of them participated in group sessions and 13 individually.	‐ Revised Children's Anxiety and Depression Scales (Chorpita et al. 2000) child and parent report to assess children's anxiety and depression symptoms ‐ Anxiety Disorders Interview Schedule for the DSMIV or DSM5 (Silverman and Albano 1996) child and parent versions were used to determine diagnostic status ‐ Clinician Global Impression Severity Scale (Guy 1976) to assess the severity of all emotional disorder diagnoses	1. Treatment 2. Longitudinal study	Anxiety and depression levels differed among children in the study. Additionally, participants in group treatment were less likely to achieve reliable changes in anxiety and depression levels compared to those in individual treatment (χ^2^ = 6.54, *p* < 0.01). Further differences emerged across severity classes. Children in the moderate severity class showed a consistent improvement (50.40%, Ms = 5.21, SE = 1.78, *p* < 0.01), while those in the high severity class exhibited rapid improvement (18.40%, Ms = 16.48, SE = 2.71, *p* < 0.001). Children in the low severity class demonstrated steady improvement (31.30%, Ms = 6.32, SE = 2.01, *p* < 0.01). The slope for the moderate severity class with consistent improvement was significantly smaller than that for the high severity class with rapid improvement (*t* = 3.47, *p* < 0.001), and the slope for the high severity class was significantly larger than the slope for the low severity class with steady improvement (*t* = 3.01, *p* < 0.01).
Kennedy et al. 2021	1. To provide an overview of the development and initial implementation of a stepped care model for delivery of the UP‐C (UP‐C‐SC), provided via telehealth, to inform future trials of stepped care delivery of transdiagnostic interventions for children. 2. Illustrate the delivery of UP‐C‐SC via three brief case examples. 3. Identify opportunities and challenges in the delivery of a transdiagnostic approach to stepped care in children.	The case studies reported are of three children: Nate: a 9‐year‐old child with separation anxiety. Regan: a 7‐year‐old child with oppositional/provocative behaviour and emotional dysregulation. Will: a 10‐year‐old child with irritability and low self‐confidence.	‐ Spence Children's Anxiety Scale child and parent version (Nauta et al. 2004) to assess anxiety symptoms ‐ Short Mood and Feelings Questionnaire (Angold et al. 1995) child and parent version were used to assess the depressive symptoms ‐ Strengths and Difficulties Questionnaire (Goodman 1997) parent report to assess behavioural difficulties and competencies of the children ‐ Paediatric Quality of Life Enjoyment and Satisfaction Questionnaire (Endicott et al. 2006) parent report to assess the quality of life	1. Treatment 2. Case study	The case studies illustrated how UP‐C‐SC can be flexibly applied to address the wide range of emotional disorder symptoms with which children commonly present, including generalized worry, adjustment symptoms, obsessions and compulsions, frustration intolerance and disruptive behaviour symptoms.
Kennedy et al. 2023	Description of the adaptation of the UP‐C programme as a treatment for delivery in a multisite general partial hospitalization programme (PHP), within a large regional children's hospital system.	Two participants: Paul, age 10, with a disruptive conduct, impulse control or unspecified disorder, ADHD. Alice, age 12, who has anhedonia and has major depressive disorder	‐ Emotional Distress–Anxiety Short Form and Depressive Symptoms Short Form (Quinn et al. 2014) to assess symptoms of anxiety and depression ‐ Emotion Dysregulation Inventor parent report reactivity Short Form (Mazefsky et al. 2018) to assess the emotion dysregulation ‐ Brief Impairment Scale (Bird et al. 2005) to assess the functional impairment	1. Treatment 2. Case study	Paul's case exemplifies how a modified version of UP‐C can effectively manage paediatric irritability and related emotional disorders, showing significant improvements in emotional reactivity and functional impairment over a 1‐month follow‐up, with anxiety and depression returning to normal levels. Conversely, Alicia's case showed minimal improvement, with worsening depressive symptoms, indicating that more intensive family work and targeted interpersonal interventions may be beneficial for her situation.
Mehrdadfar et al. 2023	1. To evaluate the effectiveness of UP‐C delivered online as a treatment for children with cochlear implantation, promoting social emotional skills. 2. To evaluate whether UP‐C for the online treatment programme influence different parent–child interactions in children with cochlear implants.	Mothers of 18 children (aged from 8 to 11 years), with cochlear implants. Of the 18 participants, 9 were randomized within the experimental group and 9 within the control group.	‐ Social Emotional Assets and Resilience Scales (Hosieni, Asemi, and Kimyaie 2015) to assess the social competence, emotion regulation, empathy and self‐esteem ‐ Children–Parent Relationship Scale (A'shouri et al. 2015) to assess parent child interaction, includes conflict, dependence and intimacy.	1.Treatment 2. Quasi‐experimental study	The results showed a difference in self‐regulation in the pretest and posttest (*F* = 23.69, *p* < 0.01) and pretest and follow‐up conditions (*F* = 6.17, *p* < 0.05). In addition, the comparison of total mean scores showed a significant difference in the pretest and posttest conditions (*F* = 9.70, *p* < 0.01). The comparison of mean scores in the different aspects of parent–child interaction in the three conditions (pretest, posttest and follow‐up) and time group interaction showed that there was a significant difference in the conflict between pretest and posttest (*F* = 5.99, *p* < 0.05) and between pretest and follow‐up (*F* = 5.99, *p* < 0.05). In addition, a significant difference was found between pretest and posttest (*F* = 6.86, *p* < 0.05) and between pretest and follow‐up (*F* = 6.72, *p* < 0.05) in dependence.
Diego et al. 2024a	1. To evaluate the short‐term effectiveness of the group version of the SSL as a treatment for children with emotional disorders. 2. To examine the benefits of SSL transdiagnostic intervention in real clinical practice.	74 children (*M*_*age*_ = 10.07, *SD* = 1.22; 71.6% boys) with internalizing disorders or trauma and/or stress‐related disorders with emotional symptoms. Out of the 74 children, 38 were in the experimental group, while 36 were in the waitlist group. Both children, and their parents (mothers: *M*_*age*_ = 42.64; *SD* = 5.30; fathers: *M* = 41.53; *SD* = 9.35), responded to assessments of children's functioning.	‐ Schedule for Affective Disorders and Schizophrenia (Endicott and Spitzer 1978) to assess the current emotional disorders ‐ Child Depression Inventory (Kovacs 1992) to assess depressive symptomology ‐ Spence Children's Anxiety Scale Parent/Child Report (Spence 1998) to assess anxiety symptoms in children ‐ Child Anxiety Life Interference Scale (Lyneham et al. 2013) child and parent report, to assess life interference and impairment related to anxiety in the child's school, social and home/family settings	1.Treatment 2. Randomized controlled trial	Significant reductions from pretest to posttest among multiple outcomes were found in children who participated in SSL compared with those in the waitlist group. Specifically, there were differences in levels of anxiety (*β* = 0.70, *p* < 0.001), trauma (*β* = 0.48, *p* < 0.001), depression (*β* = 3.39, *p* < 0.05), dysphoria (*β* = 2.30, *p* < 0.05), separation anxiety (*β* = 2.21, *p* < 0.01) and social phobia (*β* = 1.49, *p* < 0.01). As for parental measures, they reported statistically significant improvements in children's anxiety interference within the home (*β* = 2.05, *p* < 0.05).
Diego et al. 2024b	1. Examine, the effectiveness of the SSL in children with a diagnosis of mental disorder. 2. Examine changes in internalizing and externalizing symptoms before and after the intervention.	74 children (*M age =* 10.07; *SD* = 1.22; 71.6% boys) with diagnoses related to severe emotional disturbances and comorbid externalizing symptoms, as well as low self‐esteem. Out of the 74 participants, 43 were randomly assigned within the experimental group and 43 children within the waitlist group. Both children, and their parents (mothers: *M*_*age*_ = 42.64; *SD* = 5.30; fathers: *M* = 41.53; *SD* = 9.35), responded to assessments of children's functioning.	‐ Strengths and Difficulties Questionnaire (Goodman 1997) child and parent report, to assess behavioural difficulties and competencies ‐ Self‐concept Form 5 (García and Musitu 2023) to assess five dimensions of self‐concept: social, academic/professional, emotional, family and physical	1. Treatment 2. Quasi‐experimental study	At the end of treatment, children had lower levels of total difficulties (*β* = 3.01, *p* < 0.01), emotional symptoms (*β* = 1.72, *p* < 0.001) and self‐concept (*β* = 6.93, *p* < 0.01) than children on the waiting list. In addition, parents reported lower total difficulties (*β* = 3.40, *p* < 0.01), emotional symptoms (*β* = 1.35, *p* < 0.01) and problems with peers (*β* = 1.55, *p* < 0.01).
Essau and Ollendick 2019	1. To examine the effectiveness of SSL in children with emotional problems in regular school settings. 2. Investigate the change in emotional problems between preintervention and postintervention and follow‐up approximately 6 months after treatment.	205 children (*M*_*age*_ = 10.19; *SD* = 1.18, 48.29% boys) who were referred by their teachers as having significant emotional problems.	‐ Self‐Description Questionnaire (Marsh 1990) to assess the self‐concept and self‐esteem ‐ Strengths and Difficulties Questionnaire (Goodman 1997) child, parent and teachers report, to assess behavioural difficulties and competencies ‐ Screen for Child Anxiety Related Emotional Disorders (Birmaher et al. 1999) to assess the symptoms for common anxiety disorders Videos were evaluated through behavioural indicators of anxiety (Fydrich et al. 1998)	1. Prevention 2. Open clinical trial	The SSL program was effective in separation anxiety (*F* = 21.75, *p* < 0.001), panic (*F* = 12.73, *p* < 0.001), generalized anxiety (*F* = 15.50, *p* < 0.001), phobia (*F* = 7.88, *p* < 0.001) and emotional symptoms (*F* = 9.35, *p* < 0.001). In addition, parents reported a decrease in emotional symptoms (*F* = 6.86, *p* < 0.01), conduct problems (*F* = 6.22, *p* < 0.01), hyperactivity (*F* = 8.72, *p* < 0.001) and problems with peers (*F* = 7.65, *p* < 0.01). In contrast, teachers found a decrease in emotional symptoms (*F* = 9.04, *p* < 0.001) and in total difficulties (*F* = 4.01, *p* < 0.05). They also showed higher levels of self‐esteem appearance (*F* = 4.71, *p* < 0.05) and academic (*F* = 5.50, *p* < 0.01).
Fernandes et al. 2023	To examine, to what extent, executive function performance and emotion regulation at baseline would predict emotional and behavioural problem scores at postintervention SSL.	41 children (*M*_*age*_ = 9.53; *SD* = 1.09; 26.83% girls) were referred by their teachers due to observed emotional and behavioural problems.	‐ Wechsler Intelligence Scale for Children (Wechsler 1991), was used to measure working memory ‐ Strengths and Difficulties Questionnaire (Goodman 1997) to assess behavioural difficulties and competencies ‐ Cognitive Emotion Regulation Questionnaire (Garnefski et al. 2007) to assess the emotion regulation strategies ‐ Screen for Child Anxiety Related Emotional Disorders (Birmaher et al. 1999) to assess the symptoms for common anxiety disorders	1. Treatment 2. Open clinical study	The results showed a significant effect of time on strategies for adjusting emotions. The results showed that the executive functions and emotion control strategies included outcomes for emotional problems (*F* = 2.35, *p* < 0.05) and conduct problems (*F* = 2.48, *p* < 0.05). Executive functions and emotion regulation strategies also predicted hyperactivity (*F* = 2.89, *p* < 0.05), suggesting that emotion regulation strategies at preintervention significantly predicted emotional and behavioural problems after intervention. In addition, executive functions showed a significant indirect effect on both emotional problems and conduct problems, mediated by emotion regulation strategies.
Fernández‐Martínez et al. 2019	1. To examine the immediate effectiveness of SSL in reducing anxiety and depressive symptoms, selected on the basis of high scores on a measure of emotional symptoms (i.e., anxiety and depression), compared with a waitlist control group. 2. To assess whether SSL could have immediate positive effects on secondary outcome measures such as anxiety's interference with children's and parents' lives, hyperactivity, prosocial behaviour and conduct and peer problems.	123 children (*M*_*age*_ = 6.89 years; *SD* = 0.79; 44.7% girls), with emotional symptoms, and their parents. Of the 123 participants, 67 children were randomly assigned to the experimental group and 56 to the waitlist group.	‐ Mood and Feelings Questionnaire Parent version (Angold et al. 1995) to assess the depressive symptoms in their children ‐ Spence Children's Anxiety Scale Parent version (Nauta et al. 2004) to assess anxiety symptoms in their children ‐ Child Anxiety Life Interference Scale Parent report (Lyneham et al. 2013) to assess interference and impact associated with child anxiety on the life of children and their parents ‐ Strengths and Difficulties Questionnaire (Goodman 1997) to assess behavioural difficulties and competencies	1. Treatment 2. Randomized controlled trial	After the intervention, children in the SSL group showed significantly greater reductions in scores on measures of anxiety (*t* = 4.40, *p* < 0.01), fears of physical harm (*t* = 1.16, *p* < 0.01), social anxiety (*t* = 1.08, *p* < 0.05), emotional symptoms (*t* = 0.85, *p* < 0.05), anxiety interference at home (*t* = 1.14, *p* < 0.01) and depression (*t* = 3.94, *p* < 0.01) compared to children in the control group.
Fernández‐Martínez et al. 2020	1. To examine the immediate impact of SSL in improving social skills in children with subclinical anxiety symptoms. 2. Determine whether positive changes in social skills and depression were mediating factors predicting the positive impact of SSL on pre–post treatment scores in social and generalized anxiety.	123 children (*M*_*age*_ = 6.87; *SD* = 0.79; 53.2% girls). 62 children with high anxiety symptoms (the experimental group) and 56 in the control group.	‐ Mood and Feelings Questionnaire Parent version (Angold et al. 1995) to assess the depressive symptoms in their children ‐ Spence Children's Anxiety Scale Parent version (Nauta et al. 2004) to assess anxiety symptoms in their children ‐ Objective Performance Questionnaire (Cartwright‐Hatton, McNicol, and Doubleday 2003) to assess the social skills displayed during a public speech task facing a camera, rated by objective observers ‐ Social Performance Rating Scale (Fydrich et al. 1998) to assess social skills by objective coders during videotaped social situations	1. Prevention 2. Cluster randomized controlled trial	At the end of the SSL programme, there was an improvement in the scores on gaze (AOR = 1.81, *p* < 0.001), voice quality (AOR = 2.20, *p* < 0.001), length (AOR = 1.75, *p* < 0.001), discomfort (AOR = 1.67, *p* < 0.001), conversation flow (AOR = 1.31, *p* < 0.01), micro behaviours (AOR = 10.03, *p* < 0.001), nervous behaviour (AOR = 0.43, *p* < 0.001) and overall impression during speech (AOR = 7.04, *p* < 0.001). These results indicate that, after the intervention, the children significantly improved their social skills, including improvements in communication and microsocial skills and global impression, as well as reduced nervous behaviours. In addition, the reduction of depressive symptoms was the only significant mediator of change in pre–post generalized anxiety scores.
Fernández‐Martínez et al. 2020b	1. To examine the long‐term effects of SSL targeting young children with emotional problems. 2. To observe the efficacy of SSL in reducing primary outcomes, namely, anxiety and depressive symptoms. 3. To analyse the effectiveness of SSL in reducing secondary outcomes, i.e., anxiety related interference, emotional and behavioural difficulties and in increasing prosocial behaviour.	123 children (*M*_*age*_ = 6.89; *SD* = 0.79; 53.2% girls) with emotional problems. Out of the 123 participants, 67 were randomly assigned to the experimental group and 56 to the waitlist group.	‐ Spence Children's Anxiety Scale Parent version (Nauta et al. 2004) to assess anxiety symptoms in their children ‐ Mood and Feelings Questionnaire Parent version (Angold et al. 1995) to assess the depressive symptoms in their children ‐ Strengths and Difficulties Questionnaire Parent version (Goodman 1997) to assess behavioural difficulties and competencies in their children ‐ Child Anxiety Life Interference Scale—Parent report (Lyneham et al. 2013) to assess interference and impact associated with child anxiety on the life of their children	1. Prevention 2. Cluster randomized controlled trial	Participants who participated in the SSL programme, compared to those in the participants waiting list group, showed significant reductions at follow‐up in the following primary outcomes: depression (AOR = 0.01*, p* < 0.01), general anxiety (AOR = 0.002*, p* < 0.01), social phobia (AOR = 0.22*, p* < 0.05), panic/agoraphobia (AOR = 0.21*, p* < 0.01) and fears of physical injury (AOR = 0.26*, p* < 0.01). Regarding secondary outcomes, children who participated in the SSL programme, compared to those on the waiting list, showed a significant decrease at follow‐up in total difficulties (AOR = 0.08*, p* < 0.01) and emotional symptoms (AOR = 0.20*, p* < 0.001), and parents reported a decrease in general anxiety related interference in the child's life (AOR = 0.002*, p* < 0.01), both inside (AOR = 0.13*, p* < 0.05) and outside the home (AOR = 0.15, *p* < 0.05), as well as in parental life interference (AOR = 0.11*, p* < 0.05).
Fernández‐Martínez et al. 2021	1. To examine the influence that the degree of implementation fidelity (IF) may have on the effectiveness of the SSL, compared with a control group, in the short‐ and long‐term treatment in children. 2. To observe how efficacy varies by comparing high and low degrees of IF and a control group. 3. Evaluate the efficacy of IF in three dimensions of fidelity: dose, acceptance and responsiveness of participants.	123 children (*M*_*age*_ = 6.89; *SD* = 0.79; 53.2% girls), with internalizing symptoms. 26 children were randomly divided into high‐fidelity group (HFG), 41 to low‐fidelity group (LFG) and 56 to the control group	‐ Spence Children's Anxiety Scale Parent version (Nauta et al. 2004) to assess anxiety symptoms in their children ‐ Mood and Feelings Questionnaire Parent version (Angold et al. 1995) to assess the depressive symptoms in their children ‐Strengths and Difficulties Questionnaire Parent version (Goodman 1997) to assess behavioural difficulties and competencies in their children ‐ Implementation fidelity to assess acceptance dimensions, dose and responsiveness dimensions	1. Prevention 2. Randomized control trial	The SSL programme had a significant impact on depression (*β* = 5.10, *p* < 0.001), anxiety (*β* = 6.97, *p* < 0.001) and both internalizing (*β* = 2.27, *p* < 0.001) and externalizing symptoms (*β* = 0.21, *p* < 0.001). Internalizing symptoms significantly decreased only in the HFG compared to baseline and the control group. The results showed significant differences between the HFG and the control group at posttest (*p* < 0.001) and at the 12‐month follow‐up (*p* < 0.001). Externalizing symptoms significantly decreased at posttest only in the LGF (*p* < 0.01), while at the 12‐month follow‐up, they decreased only in the HFG (*p* < 0.01).
Melero et al. 2021a	1. Evaluate the effectiveness of the SSL, as a treatment in children with peer problems by promoting prosocial skills. 2. To compare the effectiveness of treatment delivered in the group mode versus the individual therapy modalities of the programme SSL.	140 children (*M*_*age*_ = 9.48; *SD* = 1.26; 35% girls) and their parents. Of these, 70 children fall into the intervention group and 70 children in the individual therapy.	‐ Strengths and Difficulties Questionnaire (Goodman 1997) child and parent version, to assess behavioural difficulties and competencies	1. Treatment 2. Quasi‐experimental study	In the posttest, children receiving individual therapy had a lower score for peer problems (AOR = 0.39, *p* < 0.001) and higher scores for prosocial behaviour (AOR = 1.84, *p* < 0.001), compared to children receiving group therapy. At 1‐year follow‐up, children in the individual therapy group had a lower score in peer problems (AOR = 0.29, *p* < 0.001) and a higher score in prosocial behaviour (AOR = 2.51, *p* < 0.001), compared to children receiving group therapy.
Melero, Morales, Espada, et al. 2021	1. To examine the short‐term effectiveness of the individual format version of the SSL in reducing symptoms of anxiety and depression. 2. To examine the impact of symptoms on the lives of children and their parents, in internalizing, externalizing difficulties and prosocial behaviour.	70 parents (*M*_*age*_ = 42.72; *SD* = 4.39) of Spanish children (*M*_*age*_ = 9.31; *SD* = 1.16; 41.4% girls), with emotional problems.	‐ Mood and Feelings Questionnaire Parent version (Angold et al. 1995) to assess the depressive symptoms in their children ‐ Spence Children's Anxiety Scale Parent version (Spence 1998) to assess anxiety symptoms in their children ‐ Child Anxiety Life Interference Scale—Parent report (Lyneham et al. 2013) to assess interference and impact associated with child anxiety on the life of their children ‐ Strengths and Difficulties Questionnaire—Parent report (Goodman 1997) to assess behavioural difficulties and competencies in their children	1. Prevention 2. Quasi‐experimental and intragroup design	The results indicate that parents observed improvements in their children's emotional state. Specifically, they identified significant reductions in symptoms of depression (*β* = 5.89, *p* < 0.001), general anxiety (*β* = 0.01, *p* < 0.001), separation anxiety (*β =* 0.15, *p* < 0.001), social phobia (*β* = 0.10, *p* < 0.001) and panic attack/agoraphobia (*β = 0.22, p* < 0.001). In addition, there was a reduction in internalizing (*β* = 0.08, *p* < 0.001) and externalizing (*β* = 5.89, *p* < 0.05) problems, as well as emotional symptoms (*β* = 0.22, *p* < 0.001), peer problems (*β* = 0.36, *p* < 0.05) and hyperactivity/inattention (*β* = 0.46, *p* < 0.05).
Melero, Orgilés, Fernández‐Martínez, et al. 2021	1. Examine the efficacy of SSL at posttest and 12‐month follow‐up according to the degree of implementation fidelity. 2. Evaluate the mediating effects of self‐concept dimensions to reduce internalizing and externalizing problems at 12‐month follow‐up.	119 children (*M*_*age*_ = 9.39; *SD* = 1.26; 42.9% girls). Out of the 119 participants, 32 were randomly divided into high‐fidelity group (HFG) and 87 to low‐fidelity group (LFG), based on the degree of implementation fidelity (IF).	‐ Strengths and Difficulties Questionnaire (Goodman 1997) to assess behavioural difficulties and competencies ‐ Children's Depression Inventory (Kovacs 1992) to assess depressive symptomology ‐ Screen for Child Anxiety Related Emotional Disorders (Birmaher et al. 1999) to assess the symptoms for common anxiety disorders ‐ Self‐Concept Form 5 (García and Musitu 2014) to assess five dimensions of self‐concept: academic, social, emotional, family and physical self‐concept ‐ Implementation Fidelity Scale to assess three dimensions: dose, adherence and acceptance	1. Treatment 2. Quasi‐experimental study	At posttest, children in the HFG scored significantly lower on depression (*z* = 2.30, *p* < 0.05) compared to the LFG, as well as higher scores in academic self‐concept (*z* = 2.49, *p* < 0.05) and physical self‐concept (*z* = 2.49, *p* < 0.05). Specifically, the HFG scored higher in depression (*r* = 0.21; *p* < 0.05) and lower in academic (*r* = 0.22; *p* < 0.01) and physical self‐concept (*r* = 0.22; *p* < 0.01). Twelve months after the programme, the HFG demonstrated superior outcomes compared to the low‐fidelity group in six out of ten measures. In summary, the SSL programme was more effective in reducing emotional symptoms in the HFG, but it also yielded positive effects in the low fidelity group, enhancing self‐concept and alleviating emotional symptoms.
Melero et al. 2022a	1. To analyse the impact of the video feedback with cognitive preparation component on the improvement of children's social performance through an objective and subjective evaluation. 2. Exploring changes in children's social behaviours.	70 children (*M*_*age*_ = 9.34; *SD* = 1.15; 29% girls), with emotional symptoms.	‐ Strengths and Difficulties Questionnaire (Goodman 1997) to assess behavioural difficulties and competencies ‐ Social Performance Rating Scale (Fydrich et al. 1998) to assess behavioural indicators of anxiety in a videotaped social performance ‐ Objective Performance Questionnaire (Cartwright‐Hatton et al. 2003) to assess micro behaviours, nervous behaviours and global impression ‐ Performance Questionnaire (Cartwright‐Hatton et al. 2003) to assess the social performance during a speech task	1. Treatment 2. Quasi‐experimental study	Regarding the change between pretest and posttest, significant improvements were found within subjects in the gaze (AOR = 3.10, *p* < 0.001), voice quality (AOR = 1.91, *p* < 0.001), length (AOR = 1.62, *p* < 0.001), discomfort (AOR = 1.99, *p* < 0.001), flow of conversation (AOR = 1.50, *p* < 0.001), in micro behaviours (AOR = 7.70, *p* < 0.001), nervous behaviours (AOR = 0.43, *p* < 0.001) and overall impression (AOR = 6.96, *p* < 0.001).
Melero et al. 2022b	1. To analyse trajectories of change in the evaluation of the SSL. 2. To assess which profile of children benefits most from the intervention and the likelihood of transition of symptom patterns over time.	119 children (*M*_*age*_ = 9.39; *SD* = 1.26; 42.9% girls), with emotional symptoms.	‐ Strengths and Difficulties Questionnaire—Parent version (Goodman 1997) to assess behavioural difficulties and competencies in their children ‐ Children's Depression Inventory (Kovacs 1992) to assess depressive symptomology ‐ Screen for Child Anxiety Related Emotional Disorders (Birmaher et al. 1999) to assess the symptoms for common anxiety disorders	1. Treatment 2. Longitudinal study	Bivariate correlations between change, depression and anxiety scores indicate significant associations between symptom reduction and higher baseline levels of panic attack disorder (*r* = 0.21, *p* < 0.05), generalized anxiety disorder (*r* = 0.23, *p* < 0.05) and social anxiety (*r* = 0.29, *p* < 0.01). A binary logistic regression showed that children with an underlying anxiety level were more likely to reduce their symptoms over time (AOR = 1.23 [1.00; 1.52], *p* < 0.05).
Orgilés et al. 2019	1. To evaluate the effectiveness of the SSL in school‐age children with internalizing symptoms. 2. To examine changes in primary outcomes, i.e., internalizing symptoms. 3. Examine changes in secondary outcomes, as well as the reduction of anxiety interference with children's lives, the reduction of symptoms of emotional and behavioural difficulties and the improvement of prosocial behaviour. 4. Evaluate changes in primary and secondary outcomes from preintervention to postintervention and preintervention, to 12‐month follow‐up.	119 children (*M*_*age*_ = 9.39; *SD* = 1.26; 42.9% girls) with internalizing symptoms.	‐ Children's Depression Inventory (Kovacs 1992) to assess depressive symptomology ‐ Screen for Child Anxiety Related Emotional Disorders (Birmaher et al. 1999) to assess the symptoms for common anxiety disorders ‐ Child Anxiety Life Interference Scale (Lyneham et al. 2013) to assess interference and impact associated with child anxiety on the life of their children ‐ Strengths and Difficulties Questionnaire (Goodman 1997) to assess behavioural difficulties and competencies	1. Treatment 2. Quasi‐experimental study	After intervention, children reported significantly lower scores for measures of depression (AOR = 0.12, *p* < 0.01), dysphoria (AOR = 0.22, *p* < 0.001), negative self‐esteem (AOR = 0.55, *p* < 0.05), generalized anxiety (AOR = 0.36, *p* < 0.05) and separation anxiety (AOR = 0.48, *p* < 0.05). In addition, children reported significantly lower scores for the interference of anxiety with the children's lives (AOR = 0.11, *p* < 0.05) both inside the home (AOR = 0.41, *p* < 0.05) and outside (AOR = 0.86, *p* < 0.05), in total difficulties (AOR = 0.33, *p* < 0.05) and emotional symptoms (AOR = 0.59, *p* < 0.05).
Orgilés, Espada, and Morales 2020	1. Describe parents' perceptions of their children's immediate psychological reactions to COVID19 quarantine, such as internalizing symptoms, externalizing symptoms and feeding and cognitive aspects and analyse the differences between the group that received SSL programme and the group who had not attended the programme. 2. Examine parents' perceptions of their children's coping styles during quarantine and analyse the differences between the SSL group and the control group.	96 parents (*M*_*age*_ = 43.50; *SD* = 5.14) of children (*M*_*age*_ = 10.52, *SD* = 2.22). Out of the 96 children, 48 belonged to the intervention group (77.1% girls) and 48 to the control group (87.5% girls).	‐ Impact Scale of the COVID19 and Home Confinement on Children and Adolescents—Parent report (Orgilés, Espada, and Morales 2020) to assess children's psychological reactions to quarantine ‐ Coping Inventory to COVID19 and Home Confinement in Children and Adolescents (Orgilés, Espada, and Morales 2020) to assess the children's coping strategies through the parents' perception	1. Prevention 2. Quasi‐experimental study	The results showed that the control group had higher levels of anxiety symptoms (*z* = 4.96, *p* < 0.001), mood problems (*z* = 3.85, *p* < 0.001), sleep problems (*z* = 2.46, *p* < 0.001) and cognitive impairments (*z* = 2.81, *p* < 0.001). Compared to coping strategies, the children in the control group sought more affection from others during confinement at home, compared to the SSL group (χ2 = 9.14; *p* < 01, OR = 2.46, 95% [IC]:1.19, 5.08).
Orgilés et al. 2020a	1. To evaluate the effectiveness at short‐term and at 12‐month follow‐up of the SSL, in children with anxiety disorders and comorbid problems. 2. To evaluate the impact of the SSL on depression, hyperactivity, conduct and peer problems, prosocial behaviour and self‐concept.	86 children (aged 8 to 12; *M*_*age*_ = 9.09; *SD* = 1.43; 60.5% boys), with separation anxiety disorder.	‐ Children's Depression Inventory (Kovacs 1992) to assess depressive symptomology ‐ Screen for Child Anxiety Related Emotional Disorders (Birmaher et al. 1999) to assess the symptoms for common anxiety disorders ‐ Strengths and Difficulties Questionnaire—Parent version (Goodman 1997) to assess behavioural difficulties and competencies in their children ‐ Self‐Concept Form 5 (García and Musitu 2014) to assess five dimensions of self‐concept: academic, social, emotional, family and physical self‐concept	1. Treatment 2. Quasi‐experimental study	Results showed that at the posttest, children had significantly lower scores in separation anxiety (AOR = 0.27, *p* < 0.001), depression (AOR = 0.27, *p* < 0.05), dysphoria (AOR = 0.30, *p* < 0.01), total difficulty (AOR = 0.28, *p* < 0.01), internalizing difficulties (AOR = 0.44, *p* < 0.05), emotional symptoms (AOR = 0.58, *p* < 0.05) and better scores for academic (AOR = 3.06, *p* < 0.01) and physical (AOR = 4.80, *p* < 0.01) measures. Twelve months after the intervention, children were given a significantly lower scores on separation anxiety (AOR = 0.07, *p* < 0.001), depression (AOR = 0.03, *p* < 0.001), dysphoria (AOR = 0.11, *p* < 0.001), negative self‐esteem (AOR = 0.30, *p* < 0.001), total difficulty (AOR = 0.04, *p* < 0.001), internalizing symptoms (AOR = 0.11, *p* < 0.001), externalizing symptoms (AOR = 0.37, *p* < 0.01), emotional symptoms (AOR = 0.31, *p* < 0.01), conduct problems (AOR = 0.51, *p* < 0.01) and peer problems (AOR = 0.35, *p* < 0.001). Instead, academic self‐concept (AOR = 6.98, *p* < 0.001), social self‐concept (AOR = 9.49, *p* < 0.001), emotional self‐concept (AOR = 6.37, *p* < 0.01) and physical self‐concept (AOR = 9.54, *p* < 0.001) were increased by 12‐months follow‐up.
Orgilés et al. 2020b	1. To evaluate the effectiveness of video feedback with cognitive preparation of the SSL, and in improving children's social performance. 2. Compare the effects of SSL on social performance behaviours. 3. To observe whether the dimensions of self and social performance prefigure as mediators of change in social anxiety and generalized anxiety.	57 children (*M*_*age*_ = 9.35; *SD* = 1.15; 68% boys) with emotional symptoms, and their parents.	‐ Self‐Concept Form 5 (García and Musitu 2014) to assess five dimensions of self‐concept: academic, social, emotional, family and physical self‐concept ‐ Screen for Child Anxiety Related Emotional Disorders (Birmaher et al. 1999) to assess the symptoms for common anxiety disorders ‐ Social Performance Rating Scale (Fydrich et al. 1998) to assess behavioural indicators of anxiety in a videotaped social performance ‐ Objective Performance Questionnaire (Cartwright‐Hatton et al. 2003) to assess micro behaviours, nervous behaviours and global impression ‐ Strengths and Difficulties Questionnaire Parent version (Goodman 1997) to assess behavioural difficulties and competencies in their children	1. Treatment 2. Quasi‐experimental study	The use of video feedback with cognitive preparation has improved children's social performance. Specifically, the children demonstrated a more appropriate tone of voice (AOR = 1.75, *p* < 0.001), adequate eye contact (AOR = 1.19, *p* < 0.01) and a friendly attitude during the second exposure (AOR = 2.28, *p* < 0.001). Therefore, children have reduced their anxiety behaviours and increased their overall social and communicative abilities. In addition, the social self‐concept was the only significant mediator of change pretest to posttest from the social anxiety (est. = 0.36, 95% CI [−0.65, −0.08]).
Orgilés et al. 2023	To examine the effectiveness of a self‐application computer programme based on the principles and objectives of SSL.	75 children (*M*_*age*_ = 9.45; *SD* = 1.3; 49.3% girls), with emotional symptoms. Of these 75 children, 35 were included in the intervention group, and 40 in the wait control list.	‐ Brief Parent Version of the Spence Children's Anxiety Scale (Reardon et al. 2018) to assess anxiety symptoms of the children ‐ Short Moods and Feelings Questionnaire (Angold et al. 1995) to assess the depressive symptoms ‐ Strengths and Difficulties Questionnaire (Goodman 1997) to assess behavioural difficulties and competencies	1. Treatment 2. Randomized controlled trial	At the end of treatment, children had lower levels of depression (*t* = 2.18, *p* < 0.01), total difficulties (*t* = 4.22, *p* < 0.01), emotional symptoms (*t* = 1.37, *p* < 0.01), hyperactivity (*t* = 1.48, *p* < 0.01), peer relationship (*t* = 1.07, *p* < 0.01), internalizing symptoms (*t* = 2.35, *p* < 0.01), externalizing symptoms (*t* = 1.69, *p* < 0.05) and anxiety (*t* = 2.65, *p* < 0.01). The parents also showed lower levels of depression (*t* = 3.51, *p* < 0.001), anxiety (*t* = 2.65, *p* < 0.001), total difficulty (*t* = 3.08, *p* < 0.001), emotional symptoms (*t* = 1.40, *p* < 0.01), conduct problems (*t* = 0.65, *p* < 0.01) and internalizing problems (*t* = 1.91, *p* < 0.01) and externalizers (*t* = 1.35, *p* < 0.01).
Ramdhonee‐Dowlot et al. 2021	1. To evaluate the effectiveness of the SSL for children with emotional problems. 2. To observe changes in primary outcomes, i.e., internalizing and externalizing symptoms, assessed at baseline, posttreatment and at 3‐month follow‐up. 3. Observe changes in secondary outcomes, i.e., in cognitive emotion regulation strategies, attentional bias and inhibitory control.	100 children and adolescents (*M*_*age*_ = 11,75; *SD* = 1,97; 76% girls). 50 children were randomly assigned to an SSL intervention group and 50 to a waitlist control group.	‐ Strengths and Difficulties Questionnaire (Goodman 1997) to assess behavioural difficulties and competencies ‐ Revised Children's Anxiety and Depression Scale (Chorpita et al. 2000) to assess children's anxiety and depression symptoms ‐ Cognitive Emotion Regulation Questionnaire (Garnefski et al. 2007) to assess cognitive emotion regulation used in response to their experience of threatening or stressful life events ‐ Rosenberg Self‐esteem Scale (Rosenberg 1965) to assess global self‐esteem	1. Prevention 2. Randomized controlled trial	After treatment, the results showed a decrease in emotional difficulties (*F* = 14.43, *p* < 0.001), conduct problems (*F* = 43.80, *p* < 0.001), hyperactivity (F = 20.15, *p* < 0.001), problems with peers (*F* = 0.18, *p* < 0.01), prosocial behaviour (*F* = 40.95, *p* < 0.001), externalizing symptoms (*F* = 57.47, *p* < 0.001), internalizing symptoms (*F* = 97.20, *p* < 0.001), total difficulties (*F* = 98.85 *p* < 0.001), generalized anxiety (*F* = 315.20, *p* < 0.001), separation anxiety (*F* = 231.00, *p* < 0.001), panic disorder (*F* = 311.60, *p* < 0.001), phobia (*F* = 120.34, *p* < 0.001), obsessive compulsive disorder (*F* = 167.87, *p* < 0.001), depression (*F* = 547.13, *p* < 0.001), self‐blame (*F* = 159.29, *p* < 0.001), rumination (*F* = 109.33, *p* < 0.001), catastrophism (*F* = 132.73, *p* < 0.001). In addition, there was better acceptance (*F* = 59.64, *p* < 0.001), in positive refocusing (*F* = 42.65, *p* < 0.001), in refocus on planning (*F* = 42.65, *p* < 0.001) and in positive reappraisal (*F* = 49.67, *p* < 0.001).

Abbreviation: *M_age_*, mean age.

### 3.1 FRIENDS Programme

Twelve articles focused on the Fun FRIENDS, FRIENDS for Life and My FRIENDS Youth. Of the 12 studies, six focused on treatment (Fjermestad et al. 2022; Fisak et al. 2018; Gallegos‐Guajardo et al. 2020; Garcia et al. 2019; Klein et al. 2021; Van Der Mheen et al. 2020), five on prevention (Hosokawa et al. 2023; Kozina 2018; Kozina 2020; Kozina 2021; Nicolaidou et al. 2021) and one addressed both treatment and prevention (Fjermestad et al. 2020). Among these, five were quasi‐experimental studies (Gallegos‐Guajardo et al. 2020; Garcia et al. 2019; Kozina 2018; Kozina 2020; Nicolaidou et al. 2021), four were RCTs (Fjermestad et al. 2020; Fjermestad et al. 2022; Klein et al. 2021; Kozina 2021), two were open trials (Fisak et al. 2018; Van Der Mheen et al. 2020) and one was a cohort study (Hosokawa et al. 2023). It was demonstrated that the transdiagnostic approach of the FRIENDS programme helped reduce internalizing symptoms in children but also produced an increase in prosocial behaviours, which is a relevant aspect and a key goal of transdiagnostic approaches (Fisak et al. 2018; Fjermestad et al. 2020; Fjermestad et al. 2022; Gallegos‐Guajardo et al. 2020; Garcia et al. 2019; Klein et al. 2021; Kozina 2018; Kozina 2020; Kozina 2021; Van Der Mheen et al. 2020). One study (Nicolaidou et al. 2021) showed that the FRIENDS programme was also helpful in decreasing obsessive‐compulsive behaviour as well as anxiety and depressive aspects. Some of the studies (Kozina 2020; Kozina 2021) on the other hand, implemented in school settings and reported promising results for the programme in preventing anxiety issues among children who had higher levels of anxiety symptoms and reduced these levels at posttreatment. However, an important aspect has also been highlighted through parental involvement in interventions as reported by some studies (Fisak et al. 2018; Klein et al. 2021).

### 3.2 Pyramid Club

Only one study (Jayman et al. 2019) was found on the Pyramid Club programme, delivered in a school setting for children aged 11–14 who participated in group sessions, reporting significant reductions in emotional symptoms and peer relationship problems and improved prosocial behaviour. This was a quasi‐experimental study focused on treatment.

### 3.3 Unified Protocol for Children (UP‐C)

Sixteen studies that were selected tested the effectiveness of the UP‐C, which has proven to be effective in counteracting internalizing and other psychological disorders. All included studies were treatment studies, and none had prevention as a goal. The studies used a variety of study designs: six were case studies (Burton et al. 2021; Eckhardt et al. 2019; Góis et al. 2022; Grossman and Ehrenreich‐May 2020; Kennedy, Lanier, et al. 2021; Kennedy et al. 2023), and two were quasi‐experimental studies (Fujisato et al. 2021; Mehrdadfar et al. 2023). The remaining studies included an RCT (Alaee et al. 2022), a randomized controlled pilot trial (Kennedy et al. 2019), a single‐arm design (Caiado et al. 2022), a semi‐experimental research (Ebrahimi et al. 2019), a qualitative study (Dimitropoulos et al. 2023), an interventional pilot study (Hawks et al. 2020), an observational study with a cohort approach (Kennedy et al. 2018) and a longitudinal study (Kennedy, Halliday, and Ehrenreich‐May 2021). In Fujisato et al.'s (2021) study, all children who participated in the study were subjects with a main diagnosis of anxiety, obsessive‐compulsive or depressive disorder, and at the end of the study, all hypotheses on the feasibility of the UP‐C with emotional disorders were supported, and symptoms decreased significantly from pretreatment to posttreatment with a large effect. In other studies (Mehrdadfar et al. 2023), the UP‐C programme has been shown to positively influence emotional perception in hearing‐impaired children. The programme was effective, and its effects were maintained for 3 months after the intervention in these subjects, who generally have an inaccurate perception of emotions and therefore experience a strong sense of frustration.

As reported by the other included studies (Alaee et al. 2022; Caiado et al. 2022; Dimitropoulos et al. 2023; Ebrahimi et al. 2019; Eckhardt et al. 2019; Fujisato et al. 2021; Góis et al. 2022; Grossman and Ehrenreich‐May 2020; Hawks et al. 2020; Kennedy et al. 2018; Kennedy et al. 2019; Kennedy, Halliday, and Ehrenreich‐May 2021; Kennedy, Lanier, et al. 2021; Kennedy et al. 2023), the results at the end of the intervention were supportive of the UP‐C programme in terms of reducing emotional problems. Children demonstrated significant reductions in anxiety and depression levels in the treated children compared to the control group. Specifically, in the study by Ebrahimi et al. (2019), a semi‐experimental research was performed, comparing UP‐C and a cognitive‐behavioural intervention. The group that underwent the intervention with the UP‐C obtained statistically significant results on anxiety and depression, achieving a significant reduction compared to the control group.

These results show that this approach can treat a range of emotional disorders (Burton et al. 2021; Eckhardt et al. 2019; Góis et al. 2022; Grossman and Ehrenreich‐May 2020; Kennedy, Halliday, and Ehrenreich‐May 2021; Kennedy, Halliday, and Ehrenreich‐May 2021; Kennedy et al. 2023). In addition, (Burton et al. 2021; Eckhardt et al. 2019) have highlighted the importance of UP‐C in dietary difficulties. In both studies, it was highlighted that the combined use of family‐based treatment and UP‐C reduced anxiety symptoms related to food intake. In other studies (Grossman and Ehrenreich‐May 2020; Hawks et al. 2020), the efficacy of UP‐C in reducing emotional symptoms related to irritability and aggression was highlighted. The results of these studies showed that at postintervention, parents had reported a reduction in the levels of irritability in children, demonstrating the effectiveness of the programme implemented.

### 3.4 SSL

Nineteen studies have focused on SSL. Of these, almost 2/3 (*n* = 12) were treatment‐based studies (Diego et al. 2024a; Diego et al. 2024b; Fernandes et al. 2023; Fernández‐Martínez et al. 2019; Melero, Morales, Espada, et al. 2021; Melero, Orgilés, Fernández‐Martínez, et al. 2021; Melero, Morales, Espada, and Orgilés 2022; Melero et al. 2021; Orgilés et al. 2019; Orgilés, Garrigós, et al. 2020; Orgilés, Melero, et al. 2020; Orgilés et al. 2023), while seven focused on prevention (Essau and Ollendick 2019; Fernández‐Martínez, Orgilés, et al. 2020; Fernández‐Martínez, Orgilés, et al. 2020; Fernández‐Martínez et al. 2021; Melero, Orgilés, Espada, and Morales 2021; Orgilés, Espada, and Morales 2020; Ramdhonee‐Dowlot et al. 2021). Eight were quasi‐experimental studies (Diego et al. 2024b; Melero, Morales, Espada, et al. 2021; Melero, Orgilés, Fernández‐Martínez, et al. 2021; Melero, Morales, Espada, and Orgilés 2022; Orgilés et al. 2019; Orgilés, Espada, and Morales 2020; Orgilés, Garrigós, et al. 2020; Orgilés, Melero, et al. 2020), five were RCTs (Diego et al. 2024a; Fernández‐Martínez et al. 2019; Fernández‐Martínez et al. 2021; Orgilés et al. 2023; Ramdhonee‐Dowlot et al. 2021) and two were cluster RCTs (Fernández‐Martínez, Morales, et al. 2020; Fernández‐Martínez, Orgilés, et al. 2020). The remaining studies employed a variety of designs, including an open clinical trial (Essau and Ollendick 2019), an open clinical study (Fernandes et al. 2023), a quasi‐experimental and intragroup design (Melero, Orgilés, Espada, and Morales 2021) and a longitudinal study (Melero, Morales, Tomczyk, et al. 2022). In several studies (Diego et al. 2024a; Diego et al. 2024b; Essau and Ollendick 2019; Fernández‐Martínez et al. 2019; Fernández‐Martínez, Morales, et al. 2020; Fernández‐Martínez, Orgilés, et al. 2020; Fernández‐Martínez et al. 2021; Melero, Morales, Espada, et al. 2021; Melero, Orgilés, Espada, and Morales 2021; Melero, Orgilés, Fernández‐Martínez, et al. 2021; Melero et al. 2022b; Orgilés et al. 2019, 2023; Ramdhonee‐Dowlot et al. 2021), positive effects of the original programme were reported, showing promising results about internalizing and externalizing symptoms as well.

The results reported by some studies (Fernández‐Martínez, Orgilés, et al. 2020; Fernández‐Martínez et al. 2021; Melero, Morales, Espada, et al. 2021; Orgilés et al. 2019) also showed significant improvements at the 12‐month follow‐up indicating that the skills learned during the programme were still being utilized 1 year after the end of the programme. In addition, another evaluation element involved video‐feedback analysis, in which behavioural aspects were considered as important indicators related to anxiety (Fernández‐Martínez, Morales, et al. 2020; Melero, Morales, Espada, and Orgilés 2022; Orgilés, Melero, et al. 2020). Results were also positive when the programme was also carried out individually (Melero, Morales, Espada, et al. 2021; Melero, Orgilés, Espada, and Morales 2021) and when it was implemented in clinical settings in computerized mode (Orgilés et al. 2023).

## 4 Discussion

In this systematic review, four of the most widely used transdiagnostic approaches for children were considered. From the selected articles, it was evident that these transdiagnostic approaches are generally effective in reducing internalizing problems but are also able to reduce externalizing problems and to improve social skills and thus prosocial behaviour. The discussion of the studies shows how transdiagnostic models leverage the importance of implementing an approach that addresses aspects that may be dysfunctional for the subject experiencing them, regardless of the diagnostic label. Before discussing the main patterns of results, two general issues are important to note. First, the majority of studies did not adopt an RCT design. Specifically, only 20.83% of all studies used an RCT methodology. Second, the vast majority of studies (*n* = 35, 72.92%) examined the programme effectiveness in treatment interventions, that is, targeting clinically‐relevant symptoms. Specifically, only FRIENDS programme had an almost equal representation of treatment and prevention studies; SSL focused on both treatment and prevention but with more studies on the former than the latter, and UP‐C included only treatment studies. Therefore, the generalizability across treatment and prevention goals requires further studies, with more evidence for the former compared to the latter. Further, it appears that the available evidence is currently provisional and wanting in support from RCT studies. Here, we first discuss the evidence that emerged from our systematic review of each of the programmes and then provide an integrative summary leading to general conclusions and recommendations.

### 4.1 The Evidence in Support of the Four Transdiagnostic Programmes

The studies reported here highlight the positive aspects and strengths of the various programmes. Regarding FRIENDS programme, the reported studies showed that FRIENDS can reduce anxiety and depression in children and highlighted the importance of implementing this programme with children for internalizing disorders (Klein et al. 2021; Kozina 2018; Kozina 2020; Kozina 2021; Nicolaidou et al. 2021). Regarding Pyramid Club programme, the only study retrieved (Jayman et al. 2019) reported a significant reduction in the difficulties specifically targeted by Pyramid (i.e., emotional symptoms and peer relationship problems), with wide‐ranging effects. This study hence provided support for Pyramid Club as an effective rogramme that targets shy, withdrawn or anxious children by aiming to improve the recipients' social–emotional skills such as confidence, self‐esteem and emotional regulation, thus strengthening resilience, although the recent evidence appears limited compared to the other approaches.

UP‐C has also been successful targeting internalizing and externalizing symptoms, addressing a wide range of aspects implicated in emotional and psychological well‐being, such as perceived control (Fujisato et al. 2021; Alaee et al. 2022). UP‐C also yielded promising results on anxiety sensitivity, and other significant results were obtained regarding the improvement of negative affectivity (Alaee et al. 2022). UP‐C is promising insofar as its creators have proposed theoretical mechanisms underlying emotional disorders. In particular, UP‐C studies have focused on the mechanisms underlying emotional regulation, which is an aspect implicated in all models of psychopathology. As evidenced by the selected studies (Burton et al. 2021; Eckhardt et al. 2019; Grossman and Ehrenreich‐May 2020), many aspects of anxiety and depression are linked to emotional regulation, and acting on it, just as the UP‐C aims to do, allows one to intervene in other basic mechanisms of psychopathology. However, the effectiveness of UP‐C does not appear to surpass that of other comparative interventions, such as cognitive‐behavioural therapy (Ebrahimi et al. 2019), family‐based treatment (Eckhardt et al. 2019) and the Cool Kids programme (Kennedy et al. 2019), in terms of remission of the primary diagnosis or overall remission of emotional disorders. This equivalence may be attributed to shared components among these treatments, such as the reduction of avoidance behaviours and the enhancement of emotion regulation skills. Consequently, the effectiveness of UP‐C might be driven by these common factors rather than by the unique features of the protocol itself.

As for the SSL programme, its results have reported satisfactory data, highlighting its effectiveness in treating internalizing but also externalizing symptomatology. A key aspect that is part of SSL is video‐feedback, which was significant in the evaluation of social performance, to change the negative evaluation children have of their social performance (Essau and Ollendick 2019; Fernández‐Martínez, Morales, et al. 2020; Melero, Morales, Espada, and Orgilés 2022; Orgilés et al. 2023). This aspect has been particularly highlighted in several studies (Fernández‐Martínez, Morales, et al. 2020; Melero, Morales, Espada, and Orgilés 2022), clarifying how the video‐feedback helped the children to reduce social anxiety and general anxiety and thus their nervous attitudes by improving their social and communication skills, guaranteeing an improvement in self‐esteem as well. Just like UP‐C, SSL also aims to intervene on emotional regulation strategies to improve resilience in young people, and such behavioural activation and cognitive reappraisal mechanisms are effective in reducing internalizing symptoms. As evidenced by the studies (Grossman and Ehrenreich‐May 2020; Kennedy et al. 2019; Orgilés, Garrigós, et al. 2020; Orgilés et al. 2023), work on rumination, negative thoughts, cognitive strategies, mindfulness and self‐esteem, led to positive effects on a wide range of internalizing and externalizing issues. Further studies (Orgilés et al. 2023; Melero, Morales, Espada, et al. 2021) have highlighted other important and innovative aspects of SSL such as the implementation of the programme on an individual level (Melero, Morales, Espada, et al. 2021; Melero, Orgilés, Espada, and Morales 2021) but also a computer level (Orgilés et al. 2023). In fact, it is now possible to carry out the programme self‐application on demand via one's own computer. These innovations are crucial because they allow benefits to be reaped by reducing costs and allowing the programme to be carried out from home.

Although the evidence for UP‐C and SSL is supported by more studies than FRIENDS and Pyramid Club, all the programmes reviewed are valid and effective in addressing emotional and behavioural issues in children, despite their use of specific and innovative approaches that foster psychological well‐being. Their effectiveness can be attributed not only to the unique features of each intervention but also to shared elements that emerge as key cross‐cutting factors. Among these, particular emphasis is placed on emotional regulation and on the reduction of avoidance behaviours.

### 4.2 Integration of Findings Across Programmes

All programmes, being transdiagnostic, consider various aspects of comorbidity and the underlying processes of the various internalizing and externalizing disorders. Therefore, in this section, the attempt is to break down the results also according to the outcomes obtained, bearing in mind that each study never focused on a single aspect, since as already discussed, transdiagnostic programmes are not designed to focus on a single disorder.

#### 4.2.1 Primary Outcomes: Anxiety and Depression

All interventions proved to be useful in reducing anxiety symptomatology; however, from the majority of the studies that emerged, SSL and UP‐C are the programmes that reported the most promising and reliable results in this respect. Although the FRIENDS programme achieved positive results about the reduction of anxiety symptoms, some studies (Kozina 2021; Nicolaidou et al. 2021) show a discordance in the achievement of these results. The Pyramid Club programme (Jayman et al. 2019) also achieved improvements in anxiety; however, more recent studies are needed to verify its effectiveness. On the other hand, SSL and UP‐C programmes were also compared with control conditions that highlighted the validity of the programme itself. For example, one study (Alaee et al. 2022) showed how the experimental group that had to do the UP‐C programme performed better on anxiety sensitivity than the control group that had done cognitive‐behavioural treatment. Other studies (Fernández‐Martínez, Orgilés, et al. 2020; Melero, Orgilés, Fernández‐Martínez, et al. 2021) showed that the fidelity of the application of the SSL programme by the experimental group produced greater positive results concerning anxiety and depression than those who had used the programme with low fidelity and about the control group. The FRIENDS programme, UP‐C and SSL have reported significant results in the reduction of separation anxiety (Gallegos‐Guajardo et al. 2020; Kennedy, Lanier, et al. 2021; Melero, Orgilés, Espada, and Morales 2021; Orgilés et al. 2019; Ramdhonee‐Dowlot et al. 2021).

The FRIENDS, UP‐C and SSL programmes have also proven to be useful for the intervention of depressive symptoms. Concerning FRIENDS, in some studies (Fisak et al. 2018; Fjermestad et al. 2020), significant results were achieved for the reduction of depressive symptoms; in another study (Van Der Mheen et al. 2020), the same results were not found. In contrast, the UP‐C and SSL programmes achieved results in the various programme applications, demonstrating their effectiveness not only at short term but also at follow‐up (Essau and Ollendick 2019; Fernandes et al. 2023; Kennedy et al. 2019).

#### 4.2.2 Secondary Outcomes

Some studies (Burton et al. 2021; Eckhardt et al. 2019) have shown that the UP‐C programme was also effective in its application with children who had avoidant and restrictive food intake disorders. Although no objective measures were used in these studies, positive effects attributable to the application of the programme were found, as patients who benefited from the UP‐C programme returned to a normal weight condition and resumed healthy eating habits. Moreover, the UP‐C programme achieved significant results to improve anger impulses and irritability (Grossman and Ehrenreich‐May 2020; Kennedy, Lanier, et al. 2021; Kennedy et al. 2023). The SSL programme has had positive results concerning externalizing disorders in children, particularly for conduct disorders (Diego et al. 2024b; Essau et al. 2014; Orgilés et al. 2023; Ramdhonee‐Dowlot et al. 2021). Furthermore, the FRIENDS programme reported a study (Gallegos‐Guajardo et al. 2020) in which prosociality was improved with increases in affective, intrapersonal and interpersonal strengths. The Pyramid Club programme showed in one study (Jayman et al. 2019) an improvement from the pretest to the posttest in prosocial behaviour in which there was also a decrease in emotional problems. UP‐C has also been shown to be effective in improving prosocial behaviour, markedly decreasing oppositional behaviour, as shown in the study by Hawks et al. (2020). Concerning SSL, statistically significant improvements in prosociality were reported in the studies of Essau and Ollendick (2019) and Melero, Morales, Espada, et al. (2021).

### 4.3 New Frontiers of Transdiagnostic Intervention: Not Only Group but Also Individual Intervention

The individual approach is relevant because it shows us that the programme, which originated at the group level, can also be administered at the individual level with excellent results in the clinical setting. The individual approach has been carried forward by FRIENDS (Klein et al. 2021), UP‐C (Burton et al. 2021; Eckhardt et al. 2019; Góis et al. 2022; Grossman and Ehrenreich‐May 2020; Kennedy, Lanier, et al. 2021; Kennedy et al. 2023) and SSL (Melero, Morales, Espada, et al. 2021; Melero, Orgilés, Espada, and Morales 2021) programmes, highlighting the innovativeness of this modality. In one study (Melero, Morales, Espada, et al. 2021), the application of the SSL programme in the group mode was compared with the individual mode, finding that children who received individual therapy presented lower scores in problems with peers and higher scores in prosocial behaviours at both the posttest and 1‐year follow‐up, compared with children who received group therapy. The UP‐C programme also emphasized the importance of conducting a study in the individual mode. Several case studies (Burton et al. 2021; Eckhardt et al. 2019; Góis et al. 2022; Grossman and Ehrenreich‐May 2020; Kennedy, Halliday, and Ehrenreich‐May 2021; Kennedy, Lanier, et al. 2021; Kennedy et al. 2023) showed their effectiveness in applying the programme in individual mode. Unfortunately, they did not include a control group.

The individual mode of the SSL programme and UP‐C was not only implemented in presence but also in computer‐based mode. The study by Orgilés et al. (2023) also highlighted the implementation of the SSL programme in the individual computer‐based mode. This mode proved effective compared to the control group: children in the experimental group presented lower scores (in both children's and parents' reports) on depressive symptoms, total difficulties, emotional symptoms, hyperactivity/disattention symptoms, peer relationship problems and internalizing/externalizing problems. In addition, the telematic modality of the UP‐C conducted in joint parent–child sessions demonstrated the effectiveness of the programme in addressing emotional problems (Kennedy, Lanier, et al. 2021; Mehrdadfar et al. 2023). This innovative application has been highly appreciated as it is a modality that manages to break down some of the barriers that may prevent children from receiving the intervention, such as distance, time, transportation and various costs (Bornheimer et al. 2018; Orgilés et al. 2023; Reardon et al. 2018).

### 4.4 Limitations

This study has some limitations to consider. Firstly, it is important to emphasize that the study was limited to a period of 6 years to look for studies that were as recent as possible. Another aspect considered in the limitations is the age range. The research focused on subjects between the ages of 4 and 14, to include the age groups investigated by the selected research programmes. Other limitations to be taken into account in the future concern the inclusion of a comparison between children and adolescents in the selected programmes, to have a greater overview according to age differences. Further, this review only considered articles written in English. A final limitation is related to the limits of the selected studies, such as the small number of RCT studies and the scant number of prevention studies compared to treatment studies.

### 4.5 Conclusions

Although transdiagnostic interventions aim to reduce emotional symptoms and internalizing disorders, they also show a positive effect on reducing externalizing symptoms and improving socioemotional skills. Individuals participating in these programmes, for example, improve their social skills, resilience and emotional regulation, which are well‐known mechanisms shared by several forms of psychopathology. Although all programmes have reported satisfactory results, the most recent studies indicate that UP‐C and SSL programmes are the most effective in reducing internalized symptoms. However, it is important to emphasize that, unlike SSL programmes, studies examining UP‐C programmes focused exclusively on treatment, without including interventions or studies aimed at prevention. This limits their applicability in preventive contexts based on the available recent evidence. However, both programmes can be applied individually and be more adaptable based on personal choices in both modalities. The strength of these transdiagnostic programmes also lies in their theory‐driven formulation and study of the processes underlying psychopathology, focusing on emotional regulation as a transdiagnostic factor that underlies various internalizing and externalizing symptoms. In the end, interventions based on the transdiagnostic approach are important as they can be flexibly targeted at a range of emotional disorders and thus also at clinical comorbidities in children (Garber and Weersing 2010). Furthermore, they are important from a cost‐effectiveness perspective as they address core processes common to several disorders within a single protocol (Craske 2012; García‐Escalera et al. 2017). Finally, the results highlight the need for more RCTs to provide stronger evidence of the efficacy of both treatment and, especially, prevention interventions, as well as the need to assess the effectiveness of programmes over longer follow‐up periods.

## Conflicts of Interest

The authors declare no conflicts of interest.

## Data Availability

Data sharing not applicable to this article as no datasets were generated or analysed during the current study.
